# Integration of stool microbiota, proteome and amino acid profiles to discriminate patients with adenomas and colorectal cancer

**DOI:** 10.1080/19490976.2022.2139979

**Published:** 2022-11-11

**Authors:** Sofie Bosch, Animesh Acharjee, Mohammed Nabil Quraishi, Irene V Bijnsdorp, Patricia Rojas, Abdellatif Bakkali, Erwin EW Jansen, Pieter Stokkers, Johan Kuijvenhoven, Thang V Pham, Andrew D Beggs, Connie R Jimenez, Eduard A Struys, Georgios V Gkoutos, Tim GJ de Meij, Nanne KH de Boer

**Affiliations:** a Department of Gastroenterology and Hepatology, Amsterdam Gastroenterology and Endocrinology Metabolism Institute, Amsterdam University Medical Centre, VU University Amsterdam, Amsterdam, The Netherlands; b College of Medical and Dental Sciences, Institute of Cancer and Genomic Sciences, Center for Computational Biology, University of Birmingham, Birmingham, UK; c Institute of Translational Medicine, University Hospitals Birmingham NHS, Foundation Trust, UK; d NIHR Surgical Reconstruction and Microbiology Research Center, University Hospital Birmingham, Birmingham, UK; e Department of Gastroenterology, University Hospitals Birmingham NHS Foundation Trust, Birmingham, UK; f Institute of Cancer and Genomic Sciences, University of Birmingham, Birmingham, UK; g Microbiome Treatment Center, University of Birmingham Microbiome Treatment Center, University of Birmingham, UK; h Center for Liver and Gastroenterology Research, NIHR Birmingham Biomedical Research Center, University of Birmingham, Birmingham, UK; i Department of Medical Oncology, Amsterdam UMC, VU University Medical Center, Amsterdam, The Netherlands; j Department of Urology, Amsterdam UMC, Cancer Center Amsterdam, Amsterdam, The Netherlands; k Institute of Applied Health Research, University of Birmingham, Birmingham, UK; l Department of Clinical Chemistry, VU University Medical Center, Amsterdam, The Netherlands; m Department of Gastroenterology and Hepatology, OLVG West, Amsterdam, The Netherlands; n Spaarne Gasthuis, Department of Gastroenterology and Hepatology, Hoofddorp and Haarlem, The Netherlands; o Microbiome Treatment Center, MRC Health Data Research UK (HDR UK), Birmingham, UK; p Microbiome Treatment Center, NIHR Experimental Cancer Medicine Center, Birmingham, UK; q Microbiome Treatment Center, NIHR Biomedical Research Center, University Hospital Birmingham, Birmingham, UK; r Department of Paediatric Gastroenterology, AG&M Research Institute, Amsterdam UMC, VU University Amsterdam, Amsterdam, The Netherlands

**Keywords:** Colon cancer, adenoma, multi omics, data integration, biomarker, stool, screening

## Abstract

**Background:**

Screening for colorectal cancer (CRC) reduces its mortality but has limited sensitivity and specificity. Aims We aimed to explore potential biomarker panels for CRC and adenoma detection and to gain insight into the interaction between gut microbiota and human metabolism in the presence of these lesions.

**Methods:**

This multicenter case-control cohort was performed between February 2016 and November 2019. Consecutive patients ≥18 years with a scheduled colonoscopy were asked to participate and divided into three age, gender, body-mass index and smoking status-matched subgroups: CRC (n = 12), adenomas (n = 21) and controls (n = 20). Participants collected fecal samples prior to bowel preparation on which proteome (LC-MS/MS), microbiota (16S rRNA profiling) and amino acid (HPLC) composition were assessed. Best predictive markers were combined to create diagnostic biomarker panels. Pearson correlation-based analysis on selected markers was performed to create networks of all platforms.

**Results:**

Combining omics platforms provided new panels which outperformed hemoglobin in this cohort, currently used for screening (AUC 0.98, 0.95 and 0.87 for CRC vs controls, adenoma vs controls and CRC vs adenoma, respectively). Integration of data sets revealed markers associated with increased blood excretion, stress- and inflammatory responses and pointed toward downregulation of epithelial integrity.

**Conclusions:**

Integrating fecal microbiota, proteome and amino acids platforms provides for new biomarker panels that may improve noninvasive screening for adenomas and CRC, and may subsequently lead to lower incidence and mortality of colon cancer.

## Introduction

Colorectal cancer (CRC) is diagnosed in over 1.8 million people each year world-wide and ranks second in terms of cancer mortality.^1,2^ Its overall 5-year survival rate is 64.4% for colon cancer and 66.6% for rectal cancer, depending on the cancer stage at diagnosis. For some adenomas, a sequence of mutations occur over a period of decades, eventually evolving into advanced adenomas.^3^ In 80% of the reported cases, colorectal carcinomas develop from initially benign colonic adenomas. As survival rate of CRC decreases gradually with increasing cancer stage at diagnosis, early detection and removal of these premalignant adenomas is crucial.^4^

Current population-based CRC screening programs aiming at selection of high-risk individuals mostly apply fecal immunochemical testing (FIT), which has been proven to reduce CRC mortality.^5^ This biomarker is, however, characterized by a limited sensitivity for both CRC (79%) and particularly for advanced adenomas (31%), leading to the performance of (unnecessary) colonoscopies which are invasive and costly.^6,7^ In addition, for a selection of CRC cases, patients are incorrectly reassured by a false-negative test.

Composition of proteins, amino acids (AA) and microbiota in stool have separately been demonstrated to hold potential as CRC biomarkers and offer the potential to be translated into easy-to-use and low-cost screening tests.^8–12^ In this study, we aimed to develop a diagnostic panel based on these omics data platforms. Second, we sought to integrate these biomarkers to obtain better insight into the interplay between the gut microbiota and metabolism in colorectal cancer and adenomas.

## Results

### Patient demographics

In total, 1093 participants collected a fecal sample of which 14 were diagnosed with CRC during endoscopy and subsequent histological examination. Two were excluded as the final histological diagnosis was a neuroendocrine tumor (NET). The 12 CRC patients (adenocarcinoma) were randomly matched on age, gender, body-mass index (BMI), and smoking status (smoker, stopped smoking or never smoked) to 21 adenoma patients (10 advanced adenomas, 11 small adenomas) and 21 controls of which one control was excluded from statistical analysis due to insufficient sample mass during the process of measurements. Detailed patients characteristics are presented in Table 1.

**Table 1.** Demographics.

	CRC (12)	AA (10)	Polyps (11)	Control (20)
Age (median [IQR])	67 [60–71]	71 [70–73]	73 [60–75]	67 [62–75]
Gender (male No [%])	6 [50]	9 [90.0]	9 [81.2]	14 [70]
BMI (median [IQR])	25.1 [23.7–31.1]	26.9 [23.5–28.4]	26.8 [23.7–29.1]	25.5 [22.9 − 28.7]
Smoking status (No[%])				
Never smoked	2 [16.7]	1 [10]	3 [27.3]	6 [13]
Stopped smoking	9 [75.0]	8 [80]	6 [44.6]	12 [60]
Actively smoking	1 [8.33]	1 [10]	2 [18.2]	2 [10]
Endoscopy indication (No [%])				
Positive FIT	6 [50]	3 [13]	3 [27.3]	3 [14]
Rectal blood loss	4 [33.3]	4 [15]	0 [0]	1 [5.0]
Abdominal pain	1 [8.33]	1 [10]	1 [9.1]	6 [13]
Diarrhea	0 [0]	0 [0]	1 [9.1]	0 [0]
Change in bowel habits	1 [8.33]	1 [10]	1 [9.1]	3 [14]
Polyp surveillance	0 [0]	1 [10]	2 [18.2]	2 [10]
Surveillance on family history	0 [0]	1 [10]	0 [0]	1 [5.0]
Incontinence	0 [0]	0 [0]	1 [9.1]	0 [0]
Coincidental radiologic finding	0 [0]	0 [0]	0 [0]	0 [0]
Surveillance after CRC	0 [0]	0 [0]	2 [18.2]	1 [5.0]
Anemia	1 [8.33]	0 [0]	0 [0]	1 [5.0]
Localization largest abnormality (No [%])*				
Cecum	0 [0]	1 [10]	2 [18.2]	NA
Ascending colon	1 [8.33]	1 [10]	3 [27.3]	NA
Flexura Hepatica	1 [8.33]	0 [0]	0 [0]	NA
Transversal colon	0 [0]	0 [0]	1 [9.1]	NA
Flexura lienalis	0 [0]	1 [10]	0 [0]	NA
Descending colon	0 [0]	0 [0]	0 [0]	NA
Sigmoid	6 [50]	4 [15]	4 [36.4]	NA
Rectosigmoid	2 [16.7]	0 [0]	0 [0]	NA
Rectum	1 [8.33]	2 [16]	1 [9.1]	NA
AA Characteristics (No [%])				
High grade dysplasia	NA	0 [0]	NA	NA
Villous histology	NA	4 [15]	NA	NA
>1 cm	NA	9 [90]	NA	NA
Polyp characteristics (No [%])				
No dysplasia	NA	NA	0 [0]	NA
Hyperplastic	NA	NA	0 [0]	NA
Low grade dysplasia	NA	NA	11 [100]	NA
Number of adenomas removed (median [IQR])	2 [1–4]	3 [2–4]	2 [1–3]	NA

Demographics of study participants. Abbreviations: CRC = colorectal cancer, AA = advanced adenoma, IQR = interquartile range, NA = not applicable. In this study, AA and polyps were combined into one adenoma group. All CRC were adenocarcinomas *Information on localization of lesion missing for one participants of the CRC and AA group.

## Multi omics data analysis

### Microbial profiles

In the present study, 2.246.463 high-quality RNA reads were obtained with a median count of 23.041 reads per sample. After taxonomic assignment, 225 operational taxonomic units (OTUs) were obtained (Supplementary Table 4). No significant differences were seen in alpha and beta diversity between the groups. The proportions of the dominant taxa were assessed at the phylum level and are depicted in bar plots in Supplementary Figure 9A-B.

When comparing CRC samples to controls, five taxa were selected from the machine learning pipeline (Supplementary Table 1). These were *Methanobrevibacter* (AUC 0.5),*Bifidobacterium* (AUC 0.78),*Eubacterium hallii* (AUC 0.64),*Ruminococcaceae UCG-003* (AUC 0.62), and *Desulfovibrio* (AUC 0.69), respectively. Combining these taxa, an AUC value of 0.78 was found (Figure 2d). Eight taxa were selected from EN and LASSO when comparing adenoma samples to controls (Supplementary Table 2). These were *Butyricimonas* (AUC 0.78), Cyanobacteria within the order of *Gastranaerophilales* with uncultured genus (AUC 0.68), *Streptococcus* (AUC 0.51),*Anaerostipes* (AUC 0.71),*Lachnospiraceae* from the FCS020 group (AUC 0.65), and ND3007 group (AUC 0.57), *Erysipelotrichaeceae* (AUC 0.62), and *Parasutterella* (AUC 0.69), respectively. A combination of these taxa resulted in an AUC value of 0.8 for the differentiation between adenomas and controls (Supplementary Figure 1D). Last, when comparing CRC samples to adenoma samples, six taxa were selected (Supplementary Table 3). These were *Butyricimonas* (AUC 0.71), Cyanobacteria within the order of *Gastranaerophilales* with uncultured genus (AUC 0.73), *Clostridialis* from the vadin BB60 group (AUC 0.73), *Tyzzerella* 3 (AUC 0.62), Firmicutes within the *Peptococcaceae* family with uncultured genus (AUC 0.68) and *Parasutterella* (AUC 0.69). Combining these six taxa, an AUC value of 0.8 was found for the discrimination between CRC and adenoma samples (Supplementary Figure 2D). Behavior of the selected taxa is visualized for each comparison in Figure 3a-c.

**Figure 1.**
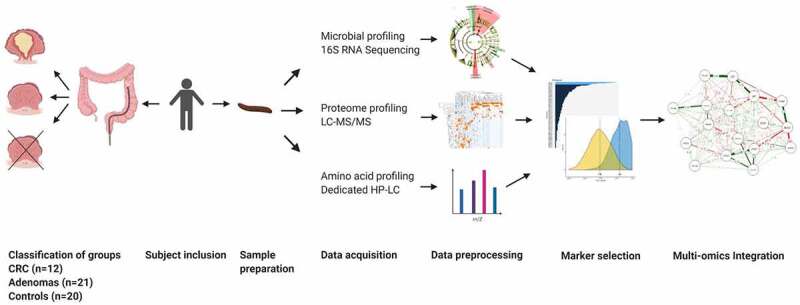
Study pipelineWorkflow of the entire study. Patients were asked to participate prior to their scheduled colonoscopy and were divided into groups: (a) colorectal cancer (CRC), (b) adenomas, (c) controls. A total of 1039 participants collected a fecal sample of which 12 were CRC. In addition, 21 adenoma and 21 controls were matched on age, body-mass index and smoking habits of which one control was excluded due to insufficient sample mass. The proteome, microbial and amino acid profiles were measured on each fecal sample. Databases were normalized. Principal component analysis was used to investigate distribution. The least absolute shrinkage selection operator and elastic net models were used to select most important markers. These markers were then combined to obtain novel accurate panels for CRC and adenoma detection. Pearson correlation was used to integrate features into a network model.

**Figure 2.**
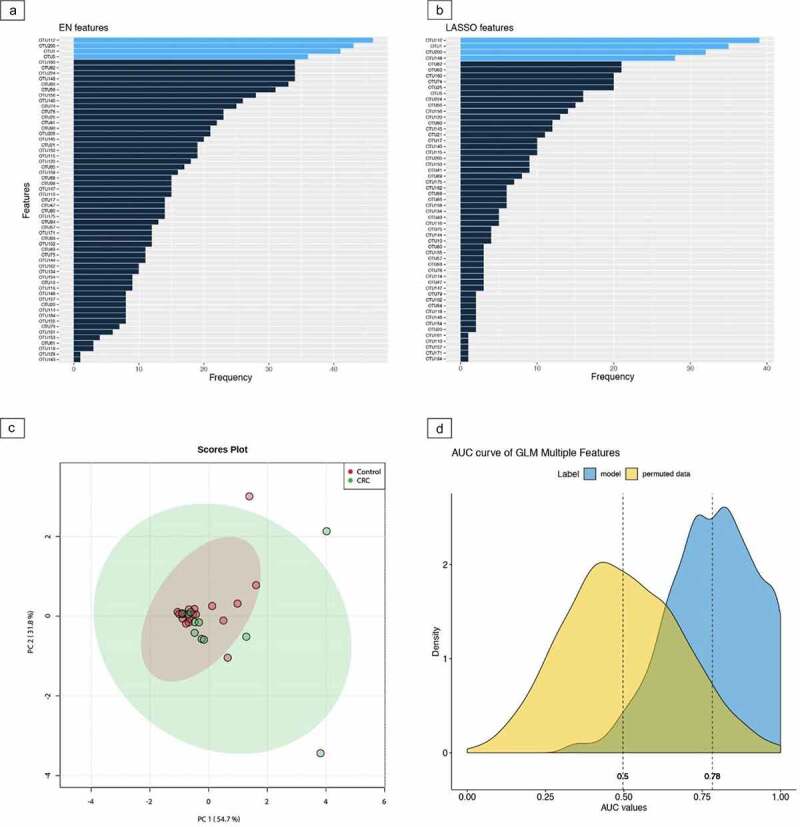
Machine learning pipeline for colorectal cancer and controls using microbial taxa The entire machine learning pipeline for the comparison between fecal samples of colorectal cancer and controls based on microbial taxa. part **a** and **b** depict the outcomes of the elastic net (EN) and least absolute shrinkage and selection operator (LASSO) feature selection methods, respectively. The light blue color in both methods indicates the first quartile of the ranked features across 100 iterations. The 5 selected markers are *Methanobrevibacter, Bifidobacterium, Eubacterium hallii, Ruminococcaceae UCG-003* and *Desulfovibrio*. In part **c**, the relatedness of the selected markers is depicted using principal component analysis (PCA). Part **d** depicts the stability plot obtained with logistic regression models for the combined marker panel that has been selected. Corresponding area under the curve (AUC) is presented in blue.

**Figure 3.**
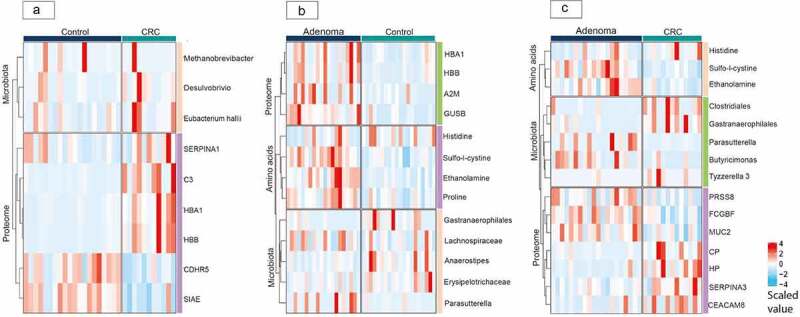
Regulation of selected markers visualized in heatmaps per comparison The heatmaps for the comparisons of fecal samples from **a**: Colorectal cancer and controls; **b**: Adenomas and controls; **c**: Colorectal cancer and adenomas. The blue-red color scale of the heatmaps depicts the level of the selected protein, microbiota and amino acid markers, in which a blue color represents a downregulation and a red color represents an upregulation. Abbreviations: CRC, colorectal cancer.

### Proteomic profiles

In total, 521 human proteins were identified from the LC-MS/MS proteomics analysis with a total median number of 169 per sample (min-max [90–281]). Based on the beta-binomial test, a total of 73 proteins differed significantly between CRC and controls, whereas 33 differed significantly between adenomas and controls and 69 proteins were significantly different between CRC and adenomas.^17^ The fold change across different samples was calculated (threshold ≥2). A list of the fold change values and corresponding proteins per comparison is given in Supplementary Tables 6–8 and the p-values of the beta-binomial test are given in the online data.

For the comparison between colorectal cancer and controls, eight proteins were selected based on the machine learning pipeline (Supplementary Table 1, Supplementary Figure 3AB). Those were SIAE (AUC 0.89), HP (AUC 0.86), CDHR5 (AUC 0.87), HBB (AUC 0.88), C3 (AUC 0.75), CP (AUC 0.84), SERPINA3 (AUC 0.76), HBA1 (AUC 0.95). Combining these eight proteins, an AUC of 0.69 was found for the discrimination between CRC and controls (Supplementary Figure 3D). When comparing adenomas to controls, the proteins GUSB (AUC 0.87), HBB (AUC 0.79), A2M (AUC 0.67) and HBA1 (AUC 0.76) were selected and had a combined AUC value of 0.87 (Supplementary Table 2, Supplementary Figure 4AB). The proteins HP (AUC 0.79), CEACAM8 (AUC 0.68), PRSS8 (AUC 0.71), MUC2 (AUC 0.75), CP (AUC 0.75), SERPINA3 (AUC 0.75), SGSH (AUC 0.75) and FCGBP (AUC 0.77) were selected for the differentiation between CRC and adenomas and a combined AUC value of 0.77 was obtained (Supplementary Table 3). Regulation of the proteomic data for all three comparisons is displayed in Figure 3a-c.

#### Protein interactions and biological processes

The selected proteins were run through the DAVID Bioinformatics and STRING consortium© databases.^14,18,19^ An overview of the selected proteins, corresponding protein interactions and biological processes is given in (Supplementary Table 5). Main associations were oxygen transport, regulation of cell death, maintenance of gastrointestinal epithelium, endocytosis, hydrogen peroxide catabolism, production of endothelial growth factor, acute phase response and inflammatory processes.

#### Validation of proteomic biomarker panels

We retrieved an online available dataset containing fecal proteomic profiles of patients with CRC (n = 79), adenomas (n = 83) and controls (n = 129).^8^ The proteomics assessment resulted in a total number of 733 features (proteins) with large overlap to our current dataset. Statistical procedure was repeated in the same manner as with our data. Results of the LASSO and EN selection methods are given in Supplementary Figure 13A-F. For the comparison between CRC and controls, five out of seven proteins from our biomarker panel were again reported as ‘most discriminative’ in either EN or LASSO analysis. Those were HP, A2M, C3, HBA, and CP. The features SIAE and HBB were selected in the validation data as important features, but not as ‘most discriminative’. For the comparison between CRC and adenomas, two out of four proteins (HBB and CEACAM8) were repeatedly selected as ‘most discriminative features’ in LASSO and EN analysis. For the comparison between adenoma and controls, different proteins were selected as most discriminative in the new validation set. Though, some of the features that appeared in our previous EN and LASSO analysis (not selected as most discriminative), did come up in the selection of EN and LASSO in the current validation data. Next, we sought for validation of our previously established test characteristics. Receiver operator characteristic curves and corresponding area under the curves for the biomarker panels as tested on the online available data are given in Figure 13 G. We were able to extract information on all proteins within the panel for the comparison between CRC and controls (C3, two forms of CP, two forms of SERPINA3, SIAE, HP, CDHR5, HBB, and HBA1), and found higher AUC value outcome compared to our study 0.842. For the comparison between adenoma and controls, we were able to select data from all proteins (HP, CEACAM8, PRSS8, MUC2, CP, SERPINA3, SGSH, and FCGBP) within the biomarker panel for the comparison between adenoma and controls. Combining these proteins gave an area under the curve for selection of adenoma patients of 0.841. For the comparison between adenomas and controls, the selected proteins A2M, HBB, HBA, and GUSB had a combined AUC of 0.51, which was lower compared to our study outcome.

### Amino acid profiles

A total number of 44 unique AA were obtained from the HPLC analysis with a median count of 26 (Interquartile range^20–25^) different AA per fecal sample. When comparing CRC samples to controls, sulfo-l-cystine (AUC 0.56), proline (AUC 0.72), and ethanolamine (AUC 0.66) were selected from the machine learning pipeline (Supplementary Table 1). Combining these AA, an AUC of 0.6 was found (Supplementary Figure 6D). For the comparison between adenomas and controls, four amino acids were selected. These were, sulfo-l-cystine (AUC 0.87), ethanolamine (AUC 0.89), proline (AUC 0.78), and histidine (AUC 0.63) (Supplementary Table 2, Supplementary Figure 7AB). An AUC of 0.89 was found when combining these AA (Supplementary Figure 7D). Sulfo-l-cystine (AUC 0.86), ethanolamine (AUC 0.80), and histidine (AUC 0.67) were selected when comparing CRC to adenoma samples and an AUC value of 0.89 was obtained when combining these proteins (Supplementary Figure 8D). Behavior of these AA is depicted for each comparison in Figure 3a-c.

### Sub-analysis advanced adenomas versus non-advanced adenoma

Four AA were selected, being ethanolamine, proline, glycine, and glutamine. Individual t-tests were not significant (p < .05) and logistic regression analysis resulted in an AUC value of 0.64. Four proteins, A2M, CEACAM1, ATIC, and C3, were selected from the machine learning pipeline and all of them significantly differed between groups when performing t-test individually (p < .05). Combining these proteins resulted in an AUC value of 0.76. Thirteen microbial taxa were selected of which nine were greatly skewed and due to sparse data were not considered further. Three taxa, *Christensenellaceae, Lachnospiraceae*, and *Ruminococcaceae* were found significant after individual t-test (p < .05). Combining these three taxa the AUC value for discriminating advanced adenomas from non-advanced adenomas was 0.65.

### Data integration for mapping of biological interactions and pathways

**Figure 4.**
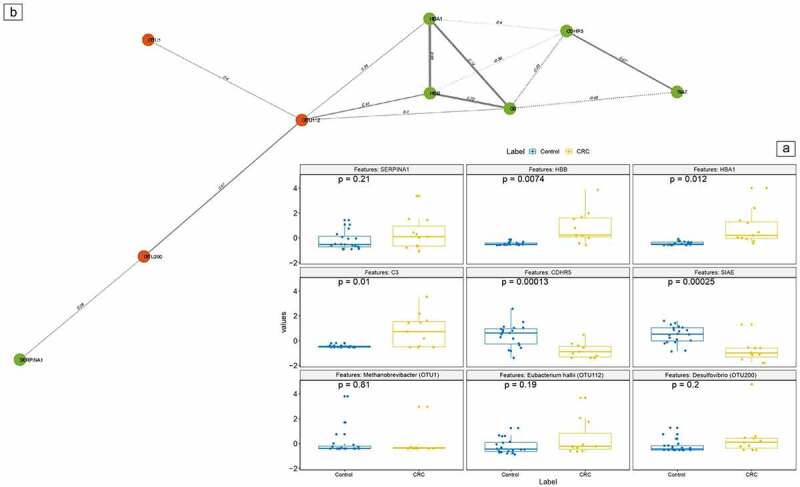
Integration network for colorectal cancer versus controls colorectal cancer versus control network. **a**: differentially expressed features of proteins, bacterial taxa and amino acids data were selected using Least Absolute Shrinkage And Selection operator (LASSO) and Elastic Net (EN). The significant correlations among the features are calculated at p < .05. **b**: Features in the boxplots correspond to the following markers (from left to right, above to below): SERPINA1, HBB, HBA1, C3, CDHR5, SIAE, *Methanobrevibacter* (OTU1),*Eubacterium hallii* (OTU112) *and Desulfovibrio* (OTU200). B: Based on Pearson correlation, selected markers from these separate datasets were combined into one integration model. In this figure, solely correlations with a coefficient above 0.3 or below −0.3 have been depicted. Each type of marker is represented as node in different colors: Proteins as green and microbial taxa as red. The correlation values are used as edge in the nodes/features. Abbreviations: CRC, colorectal cancer.

As shown in Figure 4 and Supplementary Figures 10–11, using multi-omics integration models, we observed network clusters for all three comparisons. Correlation coefficient was calculated using both Pearson, Kendall, and Spearman coefficients. Similar levels of significance were found for the selected markers and outcomes are presented in Supplementary Table 9A-C. Based on Pearson correlation analysis, significant correlations with a coefficient above 0.3 are displayed in the figures of this manuscript. We found associations between pro- and anti-carcinogenic bacteria, blood degradation products, and metabolites released in stress- and inflammatory processes, which will be further mentioned in the discussion section. All correlations per comparison are given in Supplementary Figure 12A-C.

### Selection of biomarkers for best predictive analytics

For CRC samples versus controls, three proteins stood out: SIAE, HBB, and CDHR5. Their combined AUC value was 0.98 (sensitivity 1, specificity 0.98). Comparing adenoma samples to controls, three features were selected: GUSB, Sulfo-l-cystine and ethanolamine. The combined AUC resulted in 0.95 (sensitivity 1.0, specificity 0.95). Similarly, for the comparison between CRC and adenoma samples, one AA, Sulfo-l-cystine, and one protein, HP, were selected and their combined AUC value was 0.87 (sensitivity 0.92, specificity 0.80).

### Comparison between selected biomarker panel and FIT test

As formal FIT values were not available for this dataset, the performance of the currently selected biomarker panel was compared with levels of hemoglobin as substitute, since this is the currently used protein in national population-based CRC screening programs. For this, we used all sub-units of hemoglobin available in this dataset (HBA1, HBB, HBD.HBE1, and HBG2.HBG1) and observed AUC values of 0.86, 0.81, 0.76, respectively, for CRC versus controls, adenoma versus controls and CRC versus adenomas. As described above, the newly obtained biomarker panels outperformed in accuracy compared to these hemoglobin levels in discriminating both CRC and adenomas from controls, and in discriminating CRC from adenomas.

## Discussion

In the present study, we comprehensively assessed the CRC- and adenoma-associated gut microbiota, proteome and AA composition in a case–control setting using an integrative systems biology approach. We demonstrated the complexity of their interplay in the development of CRC. In addition, we demonstrated that patients with CRC, adenomas and controls can be discriminated with high accuracy, based on a selection of features extracted from these three omics platforms.

Specific taxa, such as *Eubacterium hallii, Desulfovibrio*, and *Methanobrevibacter* displayed positive correlations with degradation products of blood particles (HBB, HBA1, and C3) and with the AA proline, leucine, and ethanolamine when comparing CRC to controls. Both microbiota and protein outcomes are in line with the previous literature.^8,9,11,26,27^ Ethanolamine metabolism has been described to play a role in carcinogenesis and tumor progression and may serve as a useful biomarker for cancer screening.^16,28^ Interestingly, we found a positive correlation between ethanolamine and the upregulated *Desulfovibrio*, which is known to ferment choline into end-products amongst which is ethanol.^29^ It may be hypothesized that upregulation of ethanolamine is due to the increased availability of ethanol in the presence of *Desulfovibrio*. Furthermore, ethanolamine is a main core membrane lipid of *Methanobrevibacter tax*a.*^30^* Upregulation of ethanolamine may possibly be due to the degradation of the upregulated *Methanobrevibacter* species, or, these species may be attracted to the colon in the presence of CRC as more ethanolamine is available in this environment. *Desulfovibrio* and *Methanobrevibacter* may both contribute to the CRC progression or presence as they are described to maintain colonic inflammation.^20,21^ Proline, an AA released during cell stress, is known to consistently contribute to tumor cell survival.^22–25^ Growth of *Eubacteria* abundance has been established on proline betaine, and attraction of *Eubacteria* to the intestines during the use of dietary proline supplements has been presented.^13^ *Eubacterium hallii* is a butyrate-producing *Eubacteria* thought to hold an anti-carcinogenic function as it detoxifies some of the most abundant dietary carcinogens into glycerol in the colon.^31^ It may be hypothesized that CRC-associated upregulation of proline contributes to the attraction of the anti-carcinogenic *Eubacterium hallii*. Comparing CRC to adenoma samples, some blood particles were still upregulated in CRC patients with CP, HP, and SERPINA3 displaying the largest difference. Positive correlation with *Tyzerrella* was found, which belongs to the class of *Clostridia*. Specific bacteria in this class have previously been associated with bloody stool itself, with and without the presence of CRC.^32,33^ Negative correlation of these blood degradation proteins with the AA histidine was found. Upregulated biosynthesis of histidine has previously been demonstrated in tissue of patients with colorectal neoplasia.^34^ Histidine metabolites have been presented to influence histamine levels, which play an important role in suppressing chronic intestinal inflammation and inflammation-associated colonic neoplasia. As a downregulation of histidine was observed in patients with adenomas, it may be hypothesized that a shortage of this AA contributes to the adenoma-carcinoma sequence at an early stage of progression. Upregulated in adenoma samples was a cluster of the proteins FCGBP, SGSH, MUC2, and PRSS8 when compared to adenomas, of which the latter two are known to play an important role in maintaining a healthy colonic epithelium. Furthermore, FCGBP has previously displayed down-regulation especially in the normal-adenoma-carcinoma sequence.^35^

The protein, GUSB, was positively correlated to upregulated blood degradation proteins in adenoma patients. GUSB degrades sulfates and upregulation in CRC tissues has previously been observed.^36^ This protein was positively correlated to sulfo-l-cysteine. The exact physiological pathway of sulfo-l-cysteine is still unknown. However, expression results in an overstimulation of glutamatergic receptors leading to calcium influx in cells.^37^ Excessive calcium influx has several consequences, among which are cytotoxicity and tissue damage. As this protein was selected in the adenoma group and not in the colorectal cancer group when compared to controls, it may be hypothesized that this cytotoxicity plays an important role in the early adenoma-carcinoma sequence.

Selecting new biomarker panels in the current study led to a high accuracy for the detection of CRC and outperformed accuracy of hemoglobin in this study, which are currently used for FIT test (0.98 versus 0.86, respectively). The selected SIAE and CDHR5 proteins are thought to play a role in maintaining colonic epithelial function and, based on our study findings, adding these proteins to the panel may improve accuracy for CRC detection in the current CRC screening program. Differentiation between adenoma and control samples, as well as between adenoma and CRC samples, was based on a combination of proteins and AA which both outperformed accuracy of hemoglobin levels in the current study (0.95 versus 0.81 for adenoma vs controls, 0.87 versus 0.76 for CRC vs adenoma). This underlines the potential to develop a noninvasive adenoma-specific screening test.

This study consisted of a prospective cohort in which cases and controls were matched on a variety of characteristics possibly influencing microbial composition and metabolomics, to prevent bias. In addition, all patients were classified according to endoscopic findings, which ensured the inclusion of control patients without any colonic abnormalities. This was the first study in which fecal protein, microbiota and AA composition were combined and simultaneously integrated to select a biomarker panel for the best prediction of CRC and adenomas. This study had several limitations which need to be addressed. The first limitation is the relatively small number of inclusions, even though measures were taken to avoid type I errors (the use of 75% training and 25% test set, 10-fold cross validation, use of machine learning methods for feature selection as well as external validation of the largest dataset), false-negative results may have occurred. Though, by providing deep phenotyping of the samples we were still able to select accurate markers and to integrate them into one highly predictive biomarker panel. Second, we validated our currently established protein panel on an existing dataset and obtained similar outcomes. However, validation of the combined omics biomarker panels was not performed, as there was no data available from previous studies covering all three omics platforms. Therefore, our current findings may be an overestimation of the accuracy in the screening population. Still, the newly established panels performed better for the detection of CRC and adenomas than the HBA1 protein in this study, which is currently used for FIT test.

In this study, we have integrated three omics platforms covering the fecal proteome, microbiota, and AA composition in patients with CRC, adenomas, and controls. Integration of data sets revealed markers associated with increased blood excretion, stress-, and inflammatory responses and pointed toward downregulation of epithelial integrity. We composed highly predictive biomarker panels consisting of proteins and AA for both CRC and adenomas detection, which outperformed accuracy of hemoglobin chains, currently used in population-based CRC screening. We were able to validate our findings on the fecal proteome in an online available cohort, in which participant selection, fecal measurements, and data processing was performed in a similar manner to our study. As most of our newly obtained biomarker panels were validated, we believe that they may improve screening for adenomas and CRC, subsequently leading to lower incidence and mortality of bowel cancer.

## Patients and methods

### Study design

Between February 2016 and November 2019, this multi-center prospective case–control study was performed at the outpatient clinics of Gastroenterology and Hepatology departments in one tertiary referral hospital (Amsterdam UMC, location VUmc, Amsterdam) and two district hospitals (OLVG West, Amsterdam and Spaarne Gasthuis, Hoofddorp and Haarlem), all located in The Netherlands. Figure 1 depicts the entire pipeline from participant inclusion to data analysis.

### Study participants and sample collection

*Detection of colorectal adenomas and cancer* Consecutive patients aged ≥18 years with a scheduled colonoscopy at one of the three hospitals were asked to participate in this study, regardless of their endoscopy indication. Based on observations during endoscopy, combined with histology reports for the cases where biopsies or polypectomies were performed, patients were divided into three subgroups: (a) CRC, histologically confirmed adenocarcinoma of the colon or rectum; (b) adenomas, including advanced adenoma, according to the European Society of Gastrointestinal Endoscopy (ESGE) guidelines (adenomas ≥1 cm in diameter, or with villous histology, or high-grade dysplasia), and including other benign adenomas defined as <1 cm, without villous histology or any grade of dysplasia lower than high-grade dysplasia;^38^ (c) controls characterized by no abnormalities observed during endoscopy (excluding hemorrhoids and/or diverticula), and where available, by no histopathological abnormalities identified in mucosal biopsies (7). Exclusion criteria were the presence of a known underlying gastrointestinal disease (e.g. inflammatory bowel disease, celiac disease), incomplete endoscopic assessment due to various reasons (e.g. hampered vision due to inadequate bowel cleansing, incomplete colonoscopy due to pain) and/or inability to collect or store sufficient fecal sample mass to perform analysis.

#### Sample and data collection

All participants collected a fecal sample (Stuhlgefäß 10 ml, Frickenhausen, Germany) prior to bowel preparation and subsequently stored the sample in their own freezer at home within one hour following bowel movement. This sample was brought to the hospital, under cooled condition, on the day of their endoscopic assessment. Samples were stored at −24°C directly upon reception. Participants completed a questionnaire which included patients demographics.

#### Endoscopic and histologic evaluation

Endoscopies were either performed or supervised by trained gastroenterologists. Endoscopy reports and histologic outcome of mucosal biopsies and/or polypectomy were assessed using the electronic patient files. The reported localization of polyps and total number of removed adenomas in this study were obtained from the endoscopy reports. Histopathological reports were used as the standard reference for size, differentiation grade of the adenomas (e.g. hyperplasia, dysplasia), villous histology and type of CRC. In the case of mucosal biopsies, size was noted as 0.2 cm. In the case multiple adenomas were present, classification was based on the most advanced or largest lesion.

### Multi-omics analysis

#### Sample preparation

For all the multi-omics analysis, frozen subsamples of 500 mg per participant were weighted and transferred into glass vials (20 ml headspace vial, Thames Restek, Saunderton, UK). Samples for amino acid analysis were transported on dry ice to the metabolic laboratory of the clinical chemistry department at the Amsterdam UMC, location VUmc. Samples for microbiota analysis were transported on dry ice to the Institute of Cancer and Genomic Sciences of the University of Birmingham (UK). For the proteomics analysis, samples were transported on dry ice to the OncoProteomics Laboratory of the department of medical oncology at Amsterdam UMC, location VUmc).

#### Amino acid analysis

By means of standard operating procedure, targeted amino acid analysis was performed on fecal samples using a targeted High Performance Liquid Chromatography (HPLC) technique, specifically amino acid analysis (AAA).^39^ The 500 mg fecal subsample and 1000 µL distilled water were mixed by vortex for one minute to homogenize the samples. The samples were then recoded and investigated by an independent laboratory researcher, blinded for the diagnosis. To prevent potential bias by differences in fecal water content, samples were frozen at minus 30 degrees and subsequently freeze-dried for 24 hours (Christ Alpha 2–4). Depending on the fecal consistency of the sample, the residual after freeze drying was approximately 30–70 mg. Consistently maintaining a feces-water ratio of 20 mg:1 mL this residual was mixed with distilled water. This mixture was again vigorously homogenized using vortex. For the analysis of the amino acid profile, 400 µL of the mixture was pipetted into a filter and centrifuged for 20 minutes at 14.000 g (Hettig Zentrifugen Mikro 2 R). Subsequently, the supernatant was mixed with an internal standard solution with a one-to-one ratio. This final mixture was centrifuged for 10 minutes and filtered (Whatman) into compatible containers for the final amino acid analyses (Biochrome 30). Amino acids were separated by ion-exchange chromatography and detected by UV-absorbance after post-column derivatization with ninhydrin.

#### Microbial 16S rRNA profiling

As part of the Qiagen AllPrep DNA/RNA Mini Kit, extracted paired DNA was used for 16S rRNA gene amplification and sequencing using the Earth Microbiome Project protocol.^15^ Using primers targeting the 16s rRNA V4 region (515 F-806 R) in a one-step, single-indexed PCR approach, the 16s rRNA genes were amplified in technical duplicates. This was done in a batch, using the appropriate negative controls. Subsequently, paired-end sequencing (2x250bp) was performed on an Illumina MiSeq platform (Illumina, San Diego, USA) and processed via the pipeline Quantitative Insights Into Microbial Ecology 2 (QIIME2).^40^ Taxonomy was assigned against the Silva-132-99% OTUs database.^41^ Relative abundances per study group were analyzed using linear discriminant analysis (LDA) effect size (LEfSe).^42^ Taxa with LDA>2 and a p-value below 0.05 were considered significant.

#### Human proteome

About 1 g of feces was weighed in at tube and was dissolved in PBS. The feces was subsequently centrifuged at 16,000 x g for 15 min at 4°C. The supernatant was collected, and centrifuged at 16,000 x g for 15 min at 4°C. The supernatant was concentrated to ~100 µl using a 3 kDa cutoff filter (Amicon, city, country). 50 µl was taken, and dissolved in LDS-sample buffer. LC-MS/MS-based proteomics analysis was performed as described previously.^8^ In brief, samples were loaded on gels (1.5mm × 10 wells). The gels were stained with Coomassie brilliant blue G-250 (Pierce, Rockford, IL) and washed and dehydrated once in 50 mM ammonium bicarbonate (ABC) and twice in 50 mM ABC/50% acetonitrile (ACN). Cysteine bonds were reduced by incubation with 10 mM DTT/50 mM ABC at 56°C for 1 h and alkylated with 50 mM iodoacetamide/50 mM ABC at RT for 45 minutes. Each sample was sliced in 1 band and further sliced up into approximately 1-mm cubes and incubated overnight at 22°C with 6.25 ng/mL trypsin (Promega, sequence grade V5111). Peptides were extracted once in 1% formic acid and twice in 5% formic acid/50% ACN. Extracted peptides were concentrated in a vacuum centrifuge (Eppendorf) to 50 µl. Peptides (5 µl) were separated on a 75 µm x 42 cm custom packed Reprosil C18 aqua column (1.9 µm, 120 Å) in a 90 min. gradient (2–32% Acetonitrile + 0.5% Acetic acid at 300 nl/min) using a U3000 RSLC high-pressure nanoLC (Dionex). Eluting peptides were measured on-line by a Q Exactive mass spectrometer (Thermo Fisher) operating in data-dependent acquisition mode. Peptides were ionized using a fused silica emitter (New Objective, Woburn MA) with a distal high voltage of +2 kV. Intact peptide ions were detected at a resolution of 35,000 (at m/z 200) and fragment ions at a resolution of 17,500 (at m/z 200); the MS mass range was 350–1,400 Da. AGC Target settings for MS were 3E6 charges and for MS/MS 2E5 charges. Peptides were selected for Higher-energy dissociation (HCD) fragmentation at an underfill ratio of 1% and a quadrupole isolation window of 1.5 Da, peptides were fragmented at a normalized collision energy of 25. Raw files from MS analysis were processed using the MaxQuant (version 1.6.4.0). MS/MS spectra were searched against the Swissprot human database (download Feb. 2019, canonical and isoforms; 42417 entries) with a precursor tolerance of 4.5 ppm and an MS/MS tolerance of 20 ppm. Peptides with minimum of seven amino-acid length were considered with both the peptide and protein false discovery rate (FDR) set to 1%. Enzyme specificity was set to trypsin and up to two missed cleavage sites were allowed. Cysteine carbamidomethylation (Cys) was searched as a fixed modification, whereas N-acetylation of proteins and oxidized methionine (Met) were searched as variable modifications (default MaxQuant settings).

### Statistical procedure

All the omics datasets sets of amino acid profiles, microbiota and proteomics data were normalized using auto scaling (mean-centered and divided by SD of each variable). The variation of each of the individual data sets was measured using principal component analysis (PCA).

#### Machine learning methods

First, we split our participants randomly into a training (75%) and test (255) dataset. Then, we used 10-fold cross validation to optimize the hyperparameters. Then, we applied two feature selection methods on the training set, Least Absolute Shrinkage and Selection operator (LASSO) and Elastic Net (EN).^43,44^ These are two forms of variable selection methods and extension of the linear regression method. Both EN and LASSO are able to automatically select the best features linked with the outcome variable from the dataset-based penalty applied and hence provide a sparse solution. Penalty parameters, λ (Range of λ:0 to 1) is optimized using 10-fold cross validation. The stronger the penalty (close to 1), smaller number of variables are selected, while if the penalty is weaker (close to 0) higher numbers of variables are selected. In other words, the penalty function λ controls the trade-off between likelihood and penalty thereby influencing the variables to be selected. The differences between regularization methods lie with the different functions they penalize. For the case of LASSO, the penalty is applied to the sum of the absolute values of the regression coefficients (L1 norm). Elastic Net, on the other hand, employs a mixed version of both L1 and L2 penalty (Ridge penalty). The L1 penalty encourages the sparse representation, whereas L2 stabilizes the solution. Similarly to LASSO, this method has an improved performance in the case that the number of features are significantly larger than the number of samples with high collinear groups of features, by allowing for grouped selection or de selection of correlated variables. We combined selected variables identified by both LASSO and EN and then applied a generalized linear model (GLM) to cater for the stability analysis of the selected features. The process was repeated 100 times and the features were ranked according to their respective selection frequency associated with each run. We then selected the first quartile from the combined LASSO and EN selected features over 100 runs. These selected features were then further modeled using logistic regression and area under the curve (AUC) calculations. We produced two AUC distributions. One is from random label sampling, i.e. randomizing the sample labels in each iteration and averaged over 100 iterations and displayed as a ‘random AUC’. The other AUC is based on the true bootstrapped samples and considered as true distributions of AUC.^45,46^

#### External validation cohort

No datasets were available online that included all three omics platforms used in this study. As our proteomics dataset consists of the highest number of features, we sought for external validation of this data and found an online available dataset in which fecal samples were processed, measured and analyzed in the same manner as to our methods.^8^

#### Network integration strategy

We applied a twostep selection approach over the different omics features. As described above, we used a machine learning pipeline to obtain best predictive markers per omics platform (amino acids, proteins, microbiota) for each of the comparisons (CRC vs. controls, adenoma vs. controls, CRC vs. adenoma). Selected markers from each data set were combined based on the comparisons and resulted in a combination of proteins, amino acids, or microbiota. A further selection was performed using machine learning methods to identify the best predictive combinations. To link the features, a Pearson correlation-based analysis on selected features (at p < .05) was performed and visualized using MATLAB. Each node represents the features (either OTUs, amino acids, or proteins), whereas the Pearson correlation value represents the edge/interaction between the features. To investigate the consistency of our data, we additionally assessed correlations using Spearman correlation and Kendall correlation coefficient.

## Supplementary Material

Supplemental Material

## Data Availability

All data on amino acid, proteomic and microbial composition generated and/or analyzed during the current study is available in the Figshare repository, and can be accessed online at 10.6084/m9.figshare.12287564. Data on patient demographics will not become publicly available due to privacy reasons.
