# Supplementary material for: Integration of stool microbiota, proteome and amino acid profiles to discriminate patients with adenomas and colorectal cancer
Source: Gut Microbes. 2022 Nov 11;14(1):2139979. doi: 10.1080/19490976.2022.2139979 (PMC9662191; doi:10.1080/19490976.2022.2139979)
Supplement: Supplemental Material [file KGMI_A_2139979_SM8477.zip › Supplementary material Figure 13AG.docx]

**Data integration of stool microbiota, proteome and amino acid profiles to discriminate patients with adenomas and colorectal cancer**

Sofie Bosch*, Animesh Acharjee*, Mohammed Nabil Quraishi, Irene Bijnsdorp, Patricia Rojas, Abdellatif Bakkali, Erwin EW Jansen, Pieter Stokkers, Johan Kuijvenhoven, Thang V Pham, Andrew D Beggs, Connie R Jimenez, Eduard A Struys, Georgios V Gkoutos, Tim GJ de Meij, Nanne KH de Boer

*shared first author

**Supplementary Figure 13: Results from external validation using online available data**

A. Feature selection using LASSO selection method for the comparison between CRC and controls using an online available proteomic dataset.


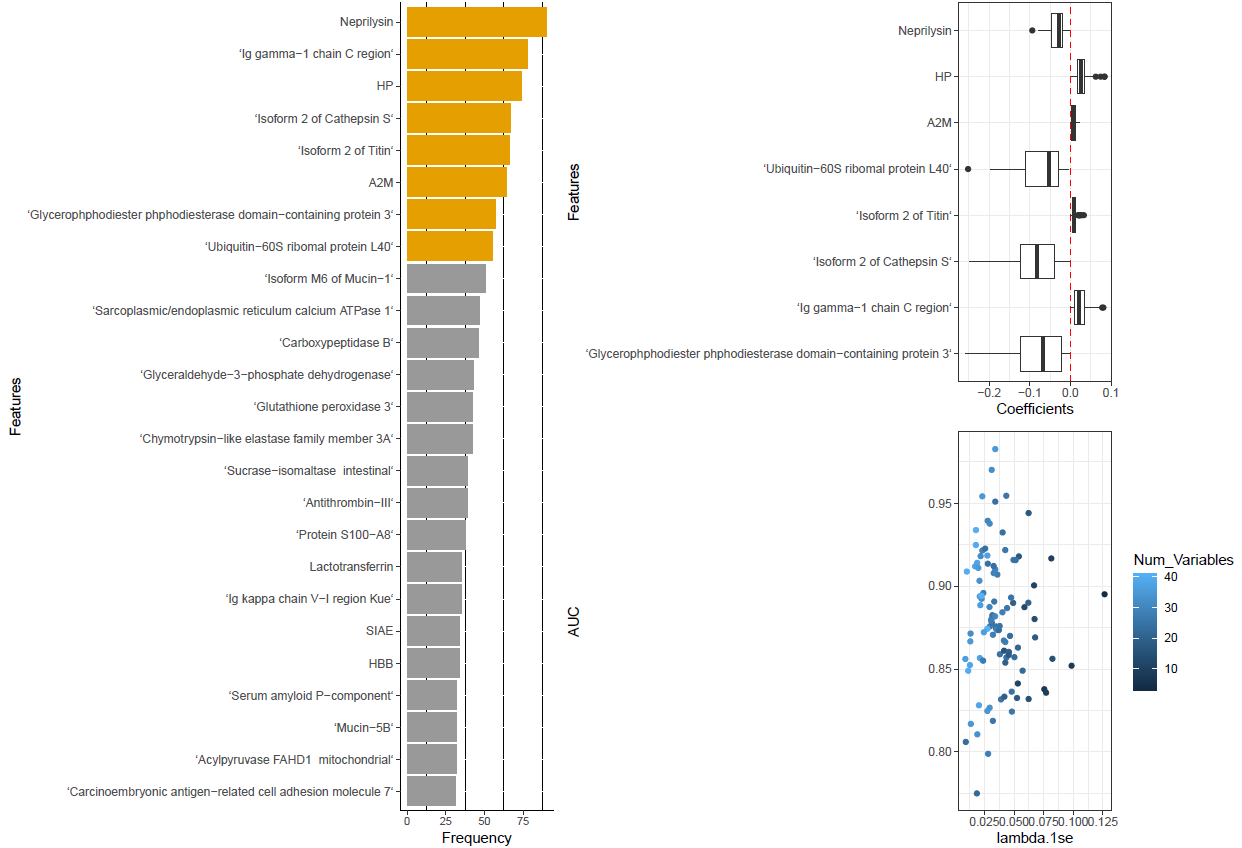


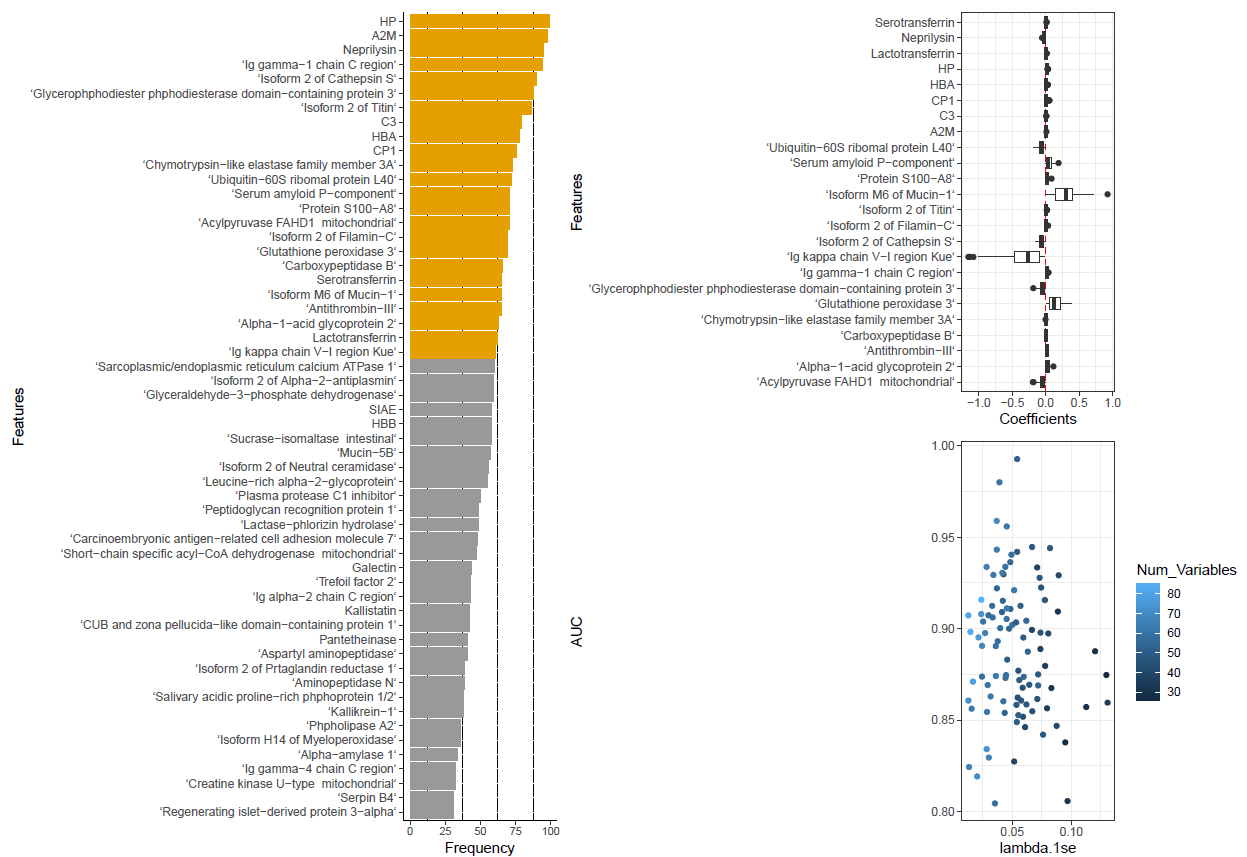
B. Feature selection using EN selection method for the comparison between CRC and controls using an online available proteomic dataset.


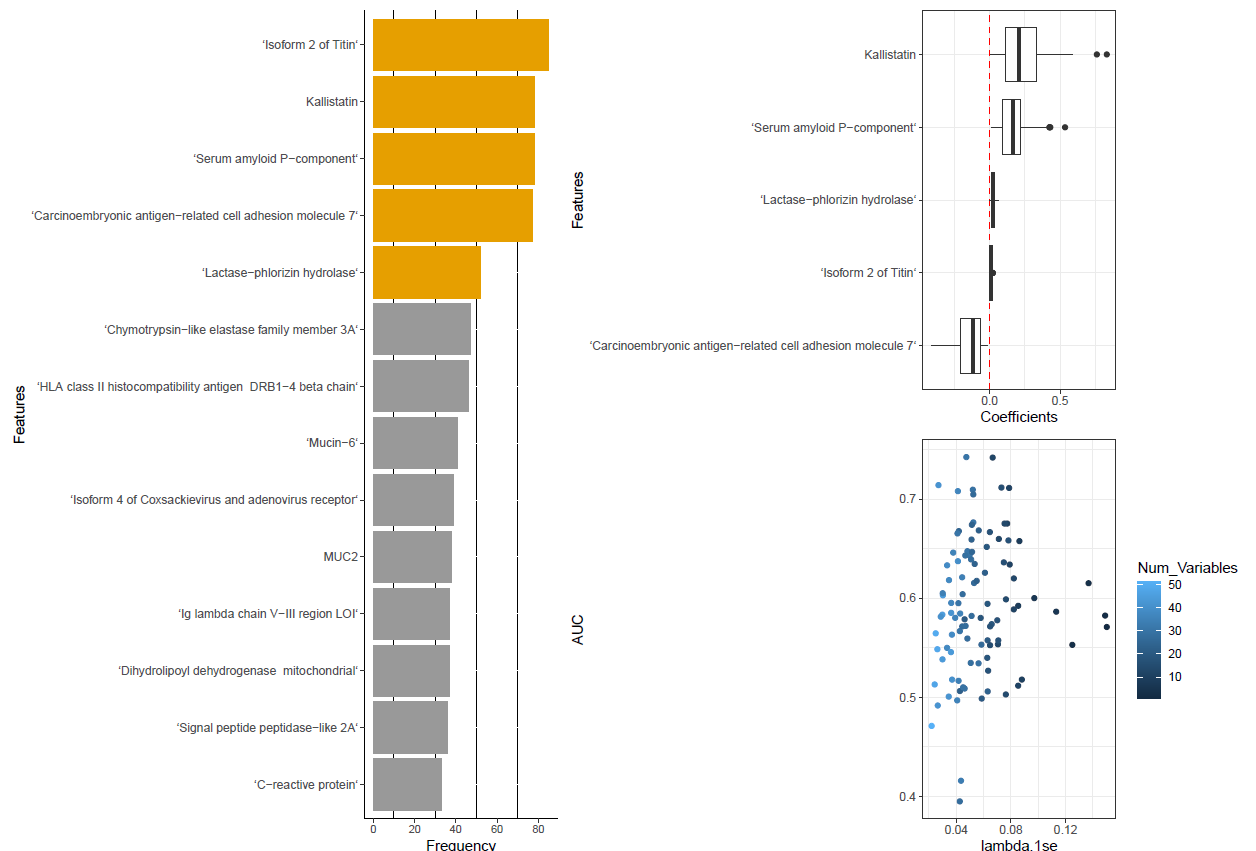
C. Feature selection using LASSO selection method for the comparison between adenoma and controls using an online available proteomic dataset.


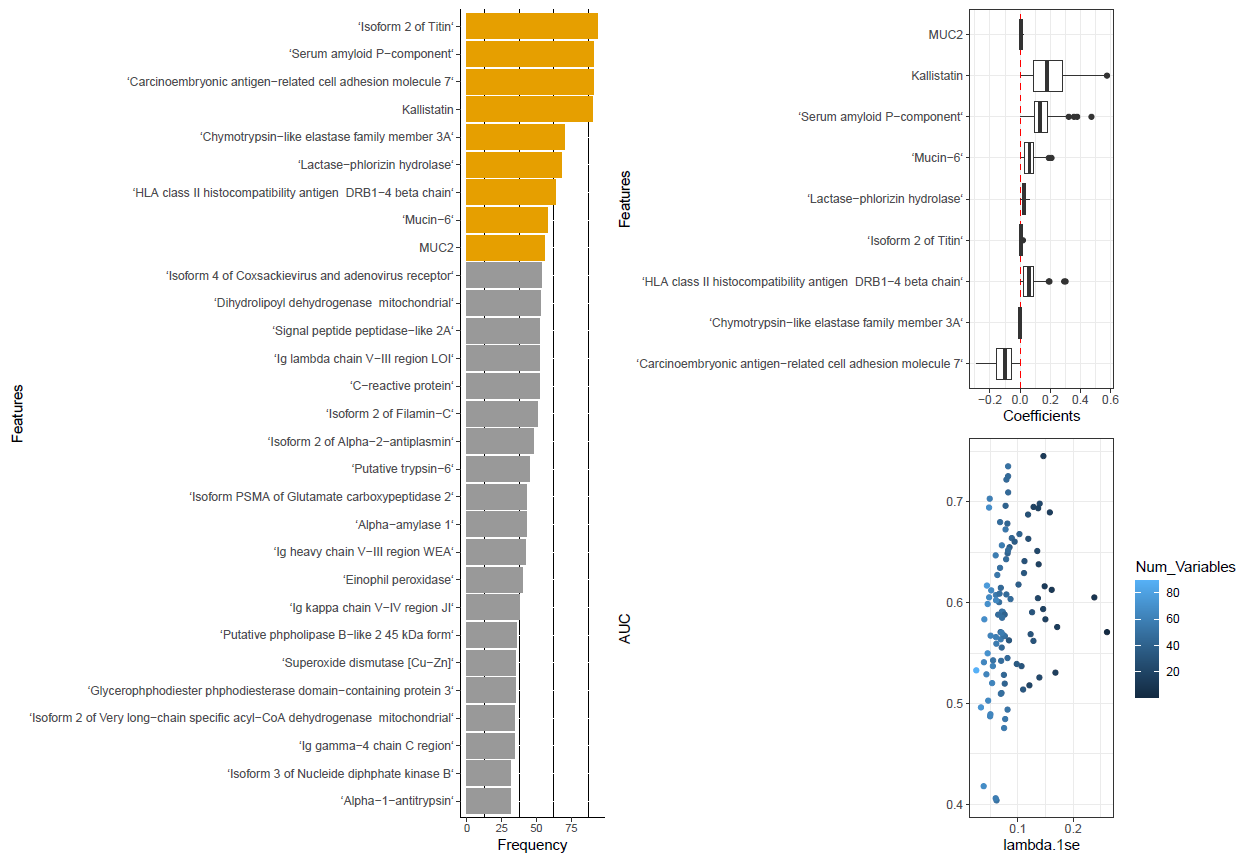
D. Feature selection using EN selection method for the comparison between adenoma and controls using an online available proteomic dataset.


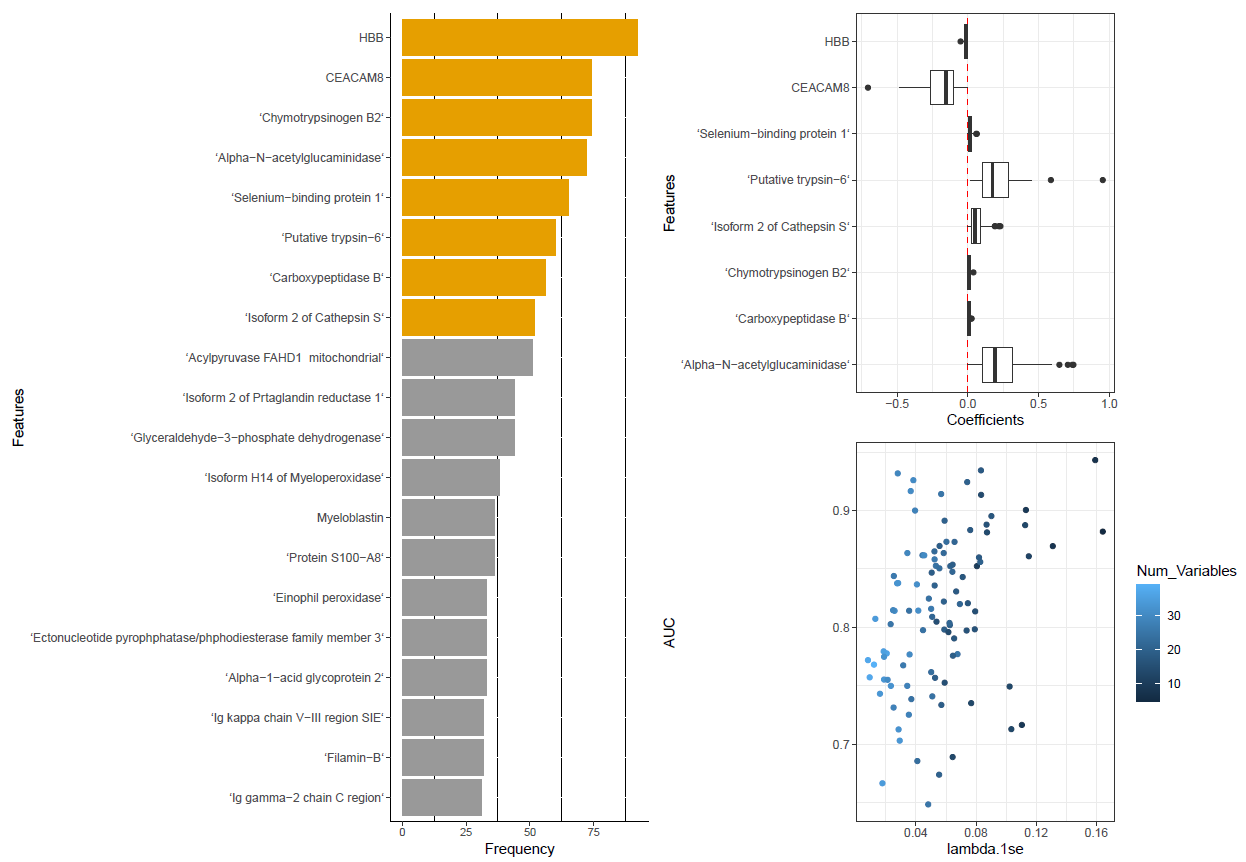
E. Feature selection using LASSO selection method for the comparison between CRC and adenoma using an online available proteomic dataset.


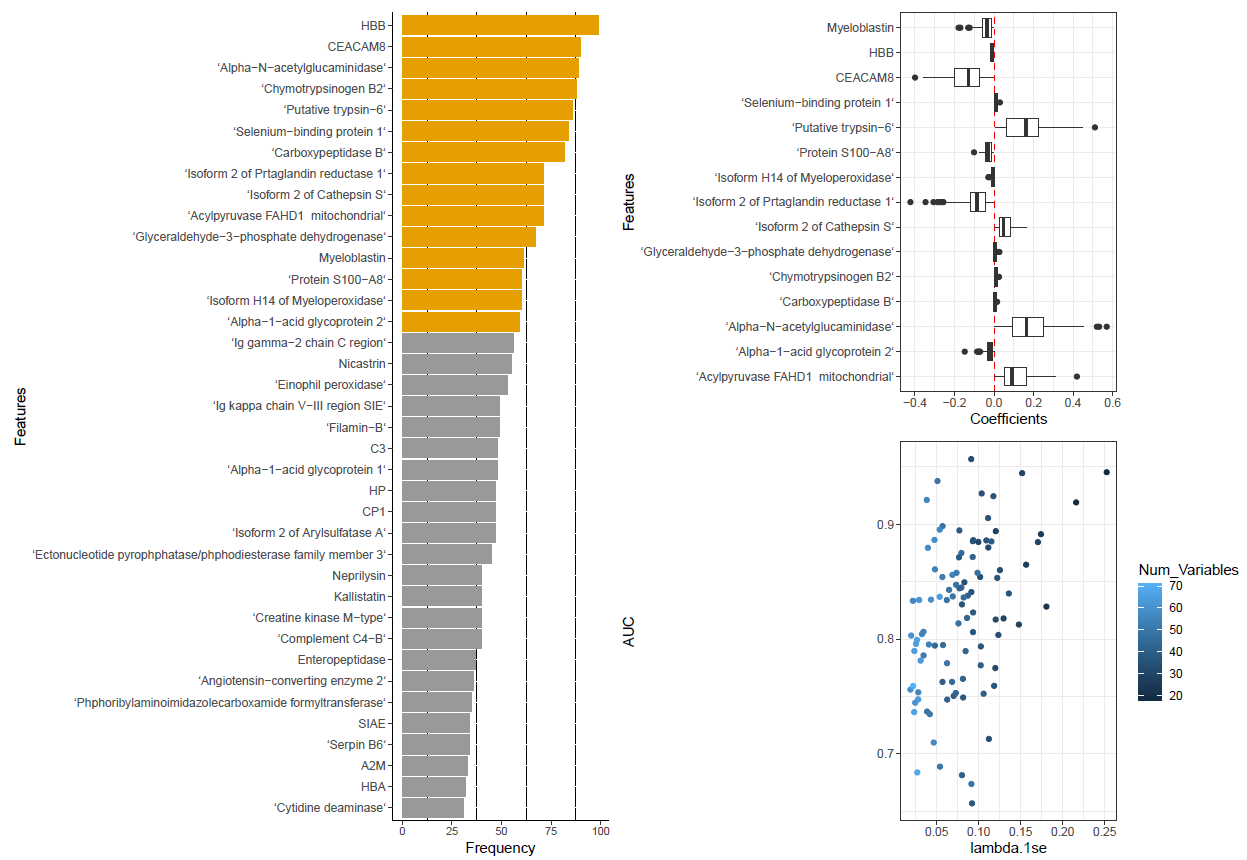
F. Feature selection using EN selection method for the comparison between CRC and adenoma using an online available proteomic dataset.


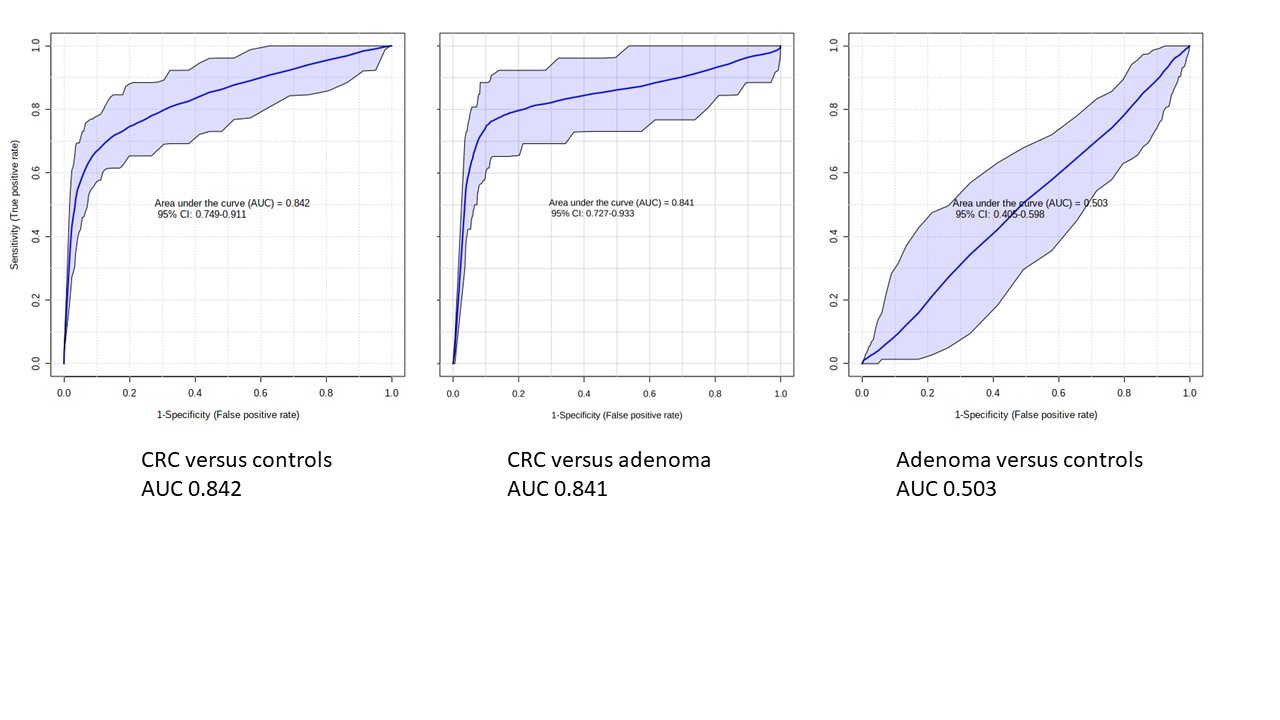
G. Receiver operator characteristic curve with corresponding area under the curve for the comparison between CRC versus control, adenoma versus control and adenoma versus CRC. Features that were selected in the current manuscript were again selected in the external online dataset when available and combined to form one biomarker panel.
