# Supplementary material for: Integration of stool microbiota, proteome and amino acid profiles to discriminate patients with adenomas and colorectal cancer
Source: Gut Microbes. 2022 Nov 11;14(1):2139979. doi: 10.1080/19490976.2022.2139979 (PMC9662191; doi:10.1080/19490976.2022.2139979)
Supplement: Supplemental Material [file KGMI_A_2139979_SM8477.zip › Supplementary Material.docx]

**Data integration of stool microbiota, proteome and amino acid profiles to discriminate patients with adenomas and colorectal cancer**

Sofie Bosch*, Animesh Acharjee*, Mohammed Nabil Quraishi, Irene Bijnsdorp, Patricia Rojas, Abdellatif Bakkali, Erwin EW Jansen, Pieter Stokkers, Johan Kuijvenhoven, Thang V Pham, Andrew D Beggs, Connie R Jimenez, Eduard A Struys, Georgios V Gkoutos, Tim GJ de Meij, Nanne KH de Boer

*shared first author

**Link to online data:** 10.6084/m9.figshare.12287564 (for referees please use the following link <https://figshare.com/s/1e775cd1b334ff4421ee> ; the DOI number will become public after publication)

**Supplementary Material Index**

[Supplementary Figure 1. Machine learning pipeline for adenoma and controls using microbial taxa 2](#_Toc42978356)

[Supplementary Figure 2. Machine learning pipeline for colorectal cancer and adenoma using microbial taxa 3](#_Toc42978357)

[Supplementary Figure 3. Machine learning pipeline for colorectal cancer and controls using proteomic profiles 4](#_Toc42978358)

[Supplementary Figure 4. Machine learning pipeline for adenoma and controls using proteomic profiles 5](#_Toc42978359)

[Supplementary Figure 5. Machine learning pipeline for CRC and adenoma using proteomic profiles 6](#_Toc42978360)

[Supplementary Figure 6. Machine learning pipeline amino acid profiles for colorectal cancer versus controls 7](#_Toc42978361)

[Supplementary Figure 7. Machine learning pipeline amino acid profiles for adenoma versus controls 8](#_Toc42978362)

[Supplementary Figure 8. Machine learning pipeline amino acids for colorectal cancer versus adenomas 9](#_Toc42978363)

[Supplementary Figure 9 Distribution of microbiota on Phylum level visualized in Bar Plots 10](#_Toc42978364)

[Supplementary Figure 10. Integration network for the comparison between adenomas and controls 11](#_Toc42978365)

[Supplementary Figure 11. Integration network for the comparison between colorectal cancer and adenomas 12](#_Toc42978366)

[Supplementary Figure 12. Integration network including all correlations per comparison 13](#_Toc42978367)

[Supplementary Table 1. Selected markers for differentiation between colorectal cancer and controls 14](#_Toc42978368)

[Supplementary Table 2. Selected markers for differentiation between adenoma and controls 15](#_Toc42978369)

[Supplementary Table 3 Selected markers for differentiation between colorectal cancer and adenoma 16](#_Toc42978370)

[Supplementary Table 4. Overview of operational taxonomic units 17](#_Toc42978371)

[Supplementary Table 5A. Overview of selected proteins and corresponding metabolic pathways 26](#_Toc42978372)

[Supplementary Table 5B. List of biological processes corresponding with selected proteins 27](#_Toc42978373)

[Supplementary Table 6. Fold change of proteins for differentiation between colorectal cancer and controls 28](#_Toc42978374)

[Supplementary Table 7. Fold change proteins for differentiation between adenomas and controls 31](#_Toc42978375)

[Supplementary Table 8. Fold change of proteins for comparison between colorectal cancer and adenomas 33](#_Toc42978376)


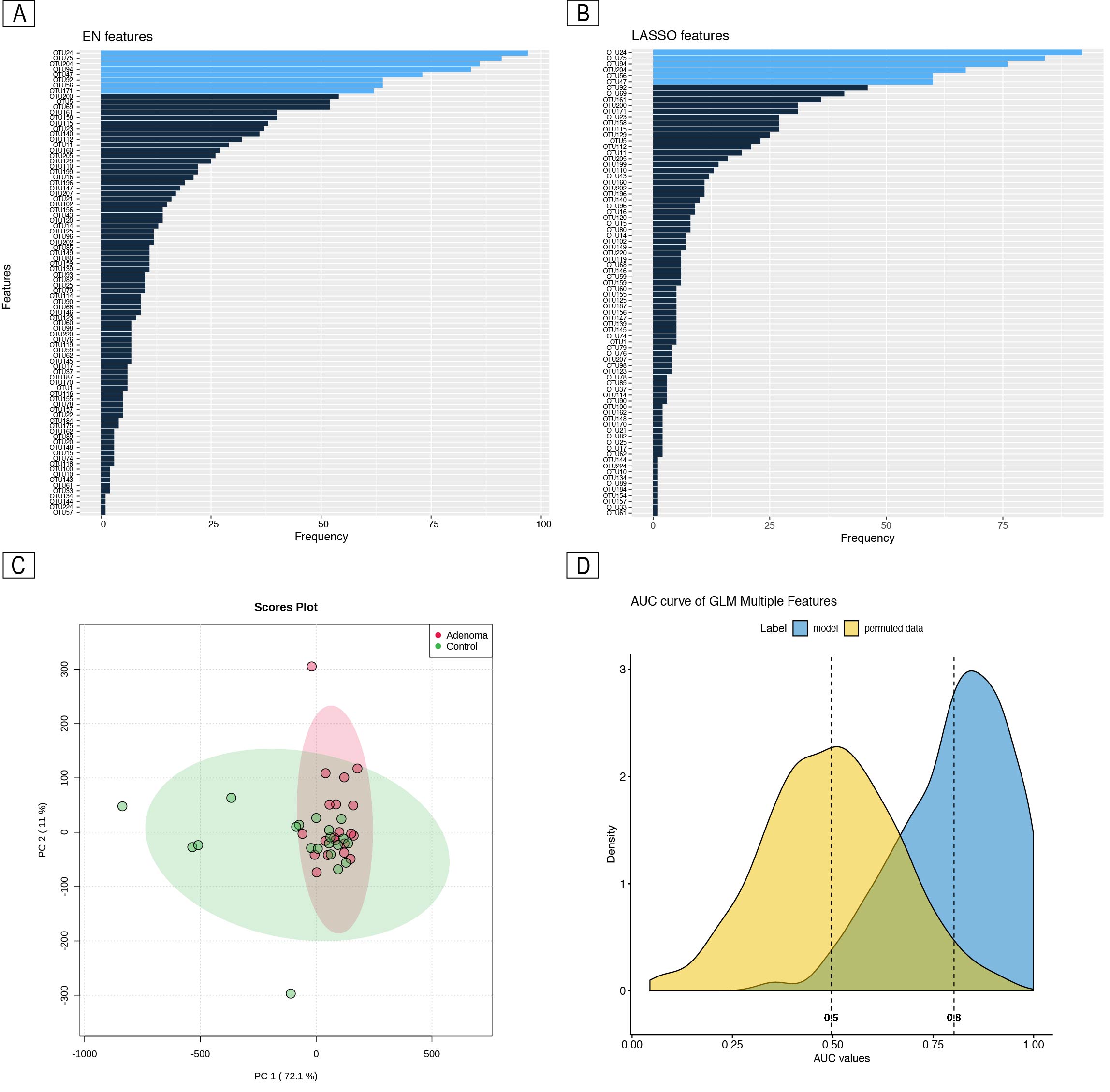
Supplementary Figure 1. Machine learning pipeline for adenoma and controls using microbial taxa

Supplementary Figure 1. The entire machine learning pipeline for the comparison between fecal samples of adenoma patients and controls based on microbial taxa. Part A and B depict the outcomes of the Elastic Net (EN) and Least Absolute Shrinkage and Selection Operator (LASSO) feature selection methods, respectively. The light blue color in both methods indicates the first quartile of the ranked features across 100 iterations. The eight selected markers were *Butyricimonas*, Cyanobacteria within the order of *Gastranaerophilales* with uncultured genus, *Streptococcus, Anaerostipes, Lachnospiraceae* from the FCS020 group and ND3007 group, *Erysipelotrichaeceae* and *Parasutterella.* In part C, the relatedness of the selected markers is depicted using Principal Component Analysis (PCA). Part D depicts the stability plot obtained with logistic regression for the combined marker panel that has been selected. Corresponding area under the curve (AUC) is presented in blue.


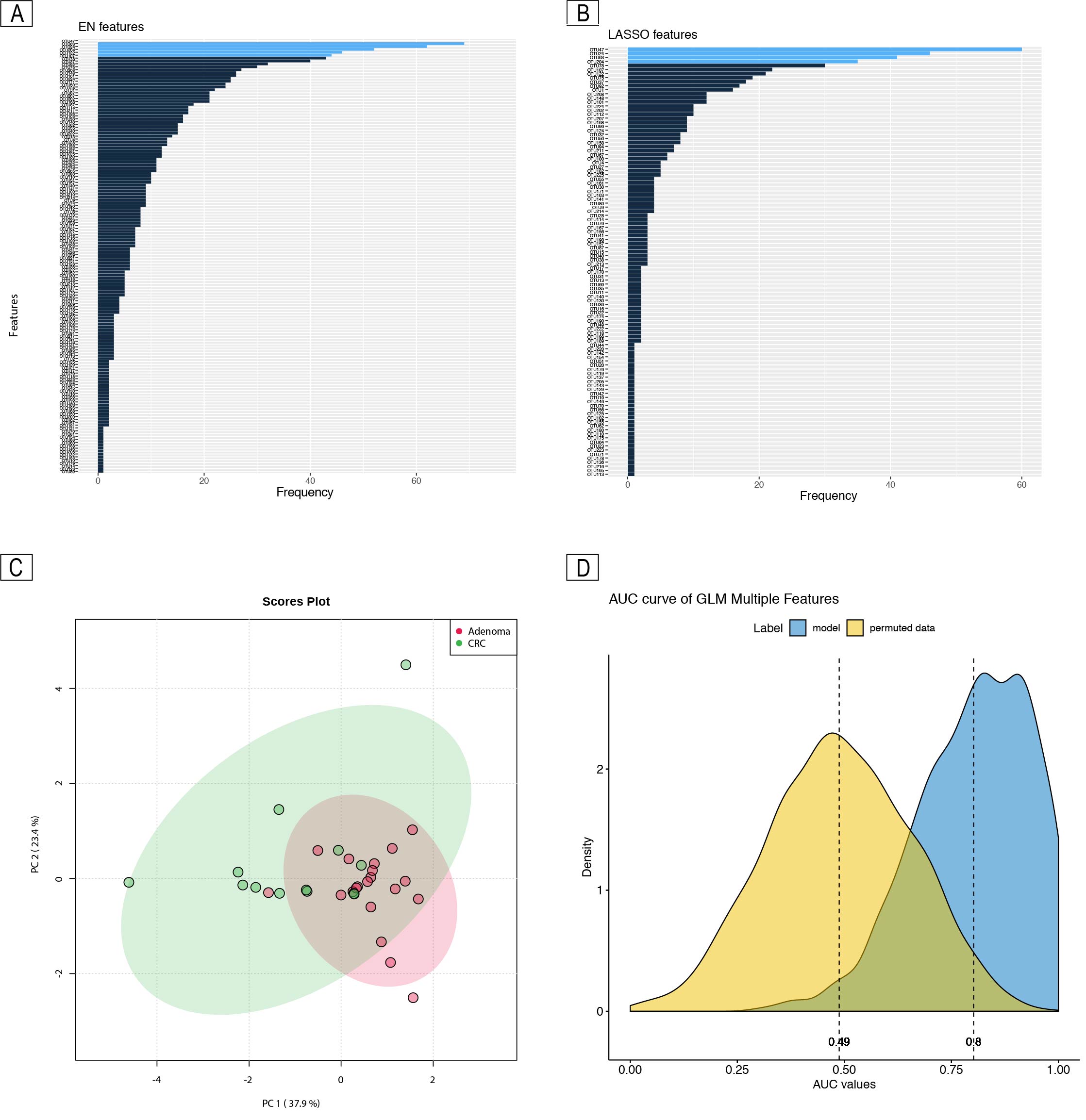
Supplementary Figure 2. Machine learning pipeline for colorectal cancer and adenoma using microbial taxa

Supplementary Figure 2. The entire machine learning pipeline for the comparison between fecal samples of colorectal cancer and adenoma patients based on microbial taxa. Part A and B depict the outcomes of the Elastic Net (EN) and Least Absolute Shrinkage and Selection Operator (LASSO) feature selection methods, respectively. The light blue color in both methods indicates the first quartile of the ranked features across 100 iterations. The six selected markers *Butyricimonas,* Cyanobacteria within the order of *Gastranaerophilales* with uncultured genus, *Clostridialis* from the vadin BB60 group, *Tyzzerella* 3, Firmicutes within the *Peptococcaceae* family with uncultured genus (AUC 0.68) and *Parasutterella*. In part C, the relatedness of the selected markers is depicted using Principal Component Analysis (PCA). Part D depicts the stability plot obtained with logistic regression for the combined marker panel that has been selected. Corresponding area under the curve (AUC) is presented in blue.


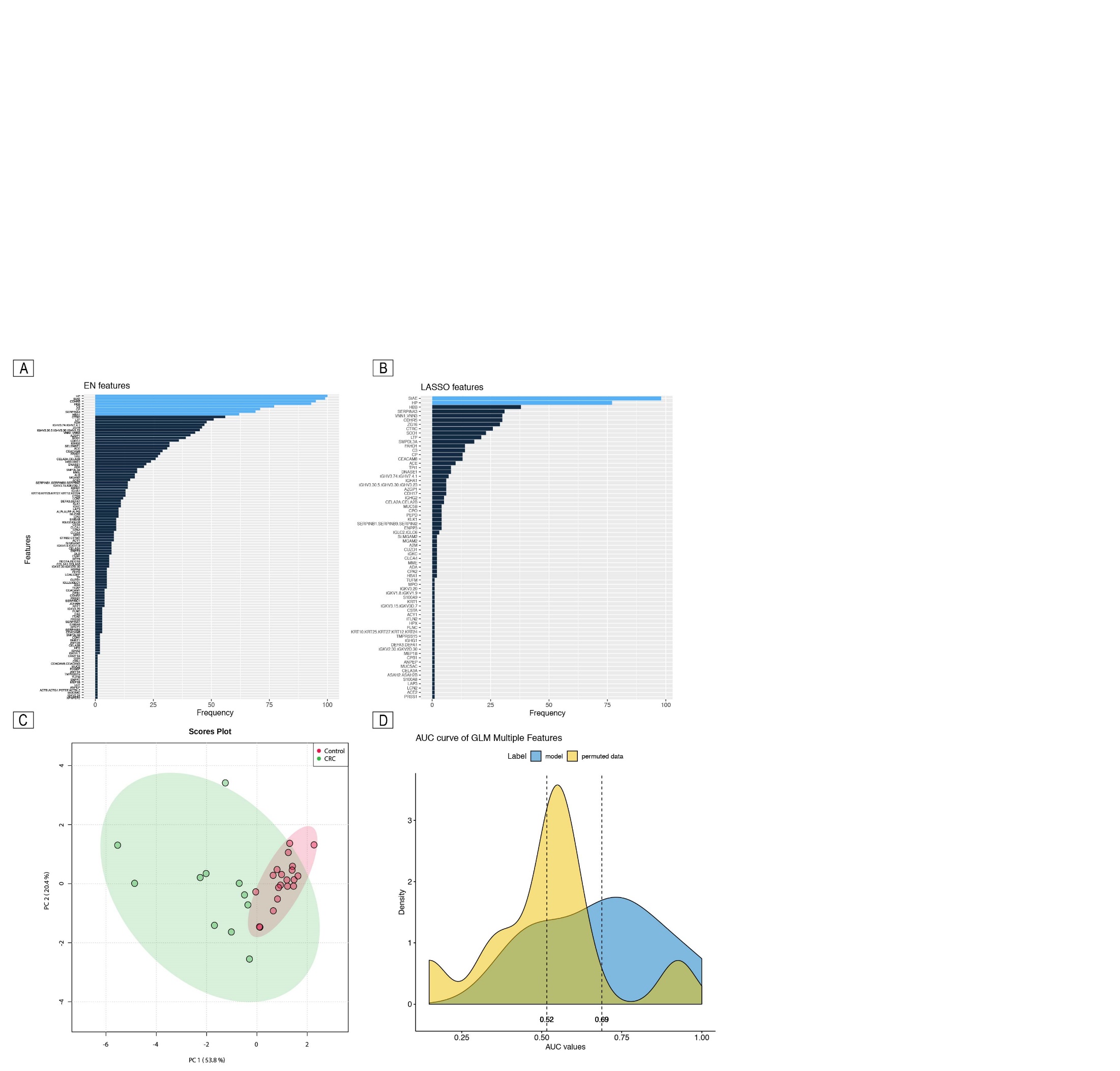
Supplementary Figure 3. Machine learning pipeline for colorectal cancer and controls using proteomic profiles

Supplementary Figure 3. The entire machine learning pipeline for the comparison between fecal samples of colorectal cancer and controls based on the proteome. Part A and B depict the outcomes of the Elastic Net (EN) and Least Absolute Shrinkage and Selection Operator (LASSO) feature selection methods, respectively. The light blue color in both methods indicates the first quartile of the ranked features across 100 iterations. Selected proteins were SIAE, HP, CDHR5, HBB, C3, CP, SERPINA3, HBA1. In part C, the relatedness of the selected markers is depicted using Principal Component Analysis (PCA). Part D depicts the stability plot obtained with logistic regression for the combined marker panel that has been selected. Corresponding area under the curve (AUC) is presented in blue.


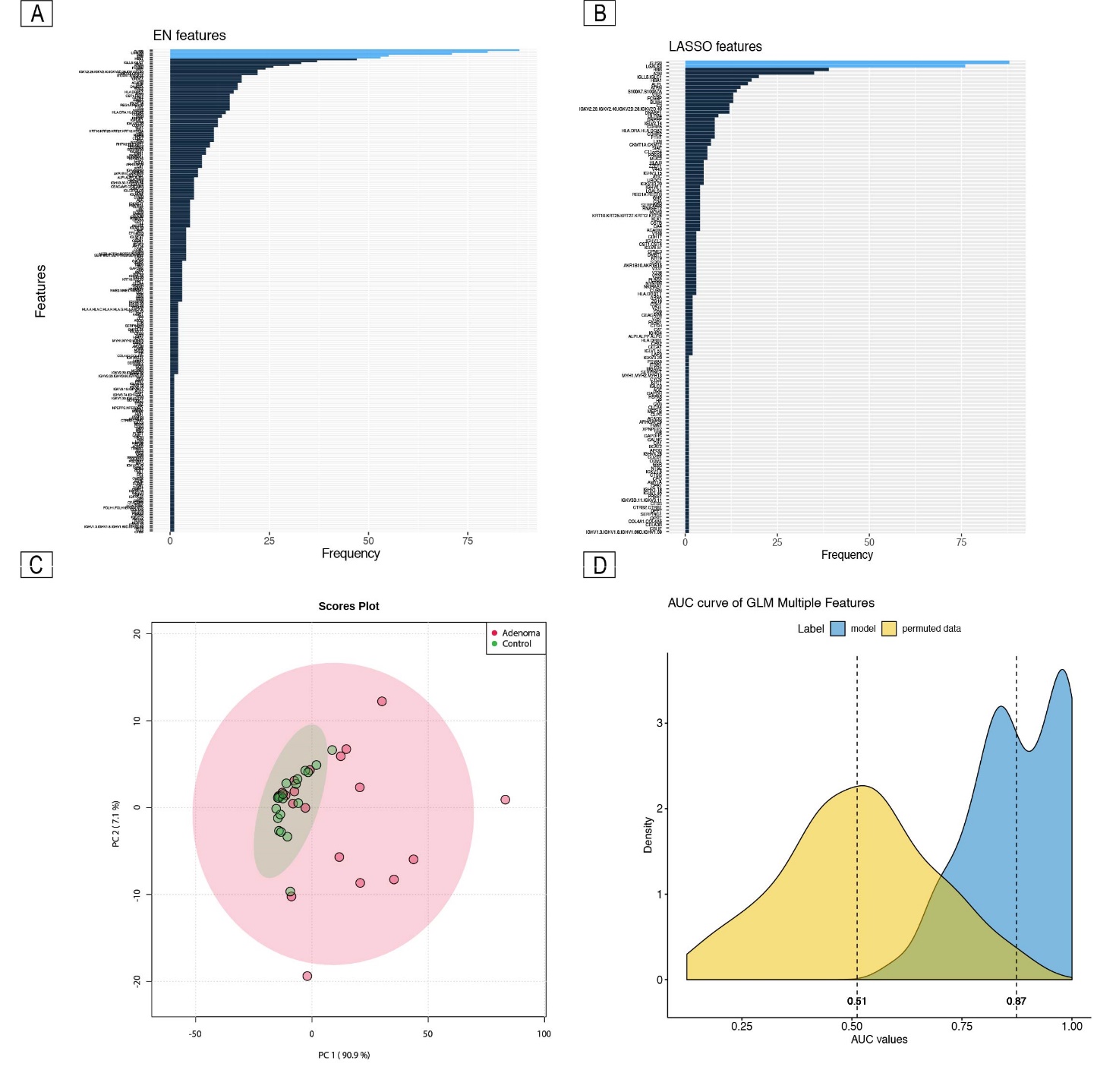
Supplementary Figure 4. Machine learning pipeline for adenoma and controls using proteomic profiles

Supplementary Figure 4. The entire machine learning pipeline for the comparison between fecal samples of adenoma patients and controls based on the proteome. Part A and B depict the outcomes of the Elastic Net (EN) and Least Absolute Shrinkage and Selection Operator (LASSO) feature selection methods, respectively. The light blue color in both methods indicates the first quartile of the ranked features across 100 iterations. Selected proteins were GUSB, HBB, A2M and HBA1. In part C, the relatedness of the selected markers is depicted using Principal Component Analysis (PCA). Part D depicts the stability plot obtained with logistic regression for the combined marker panel that has been selected. Corresponding area under the curve (AUC) is presented in blue.


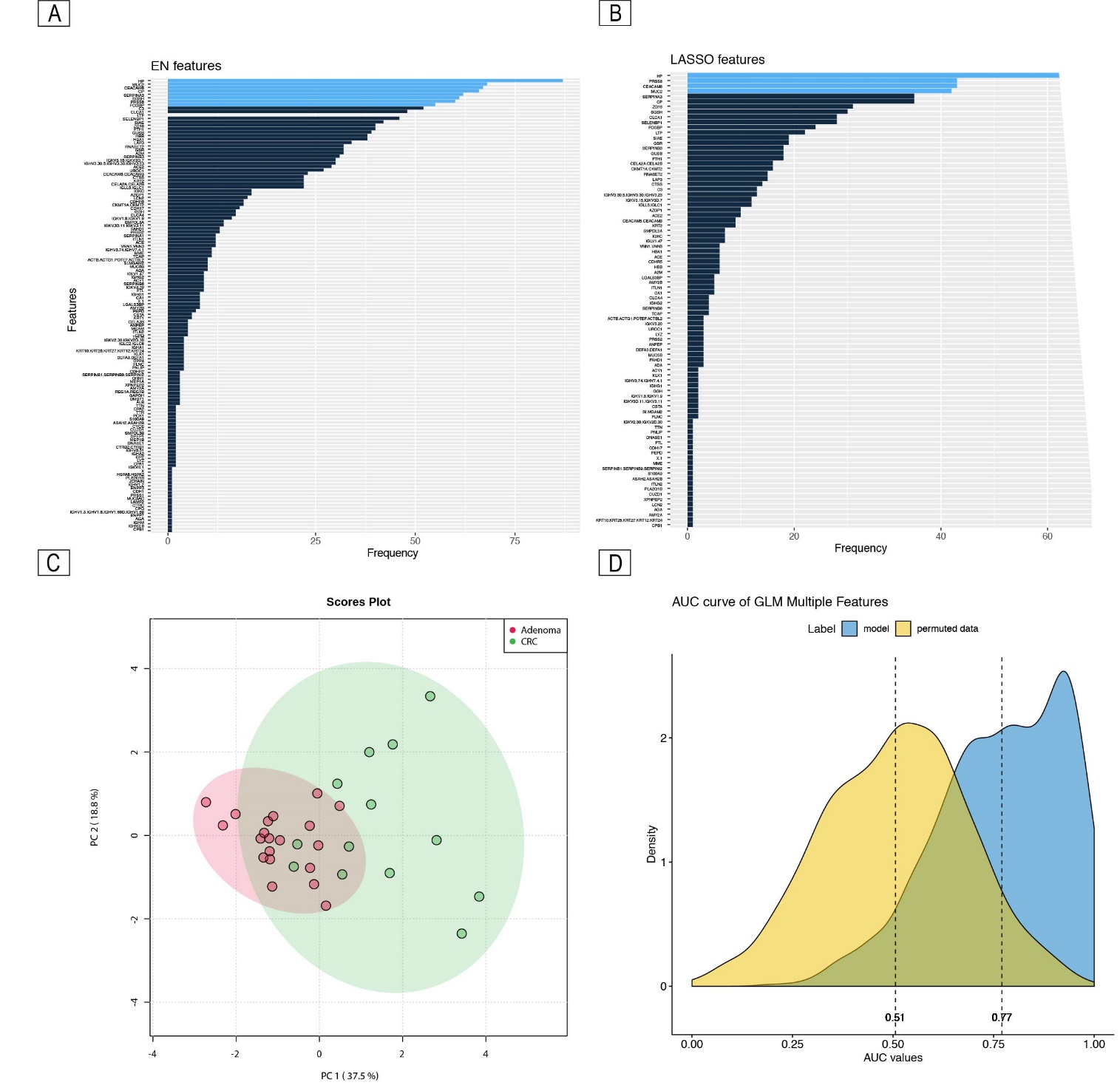
Supplementary Figure 5. Machine learning pipeline for colorectal cancer and adenoma using proteomic profiles

Supplementary Figure 5. The entire machine learning pipeline for the comparison between fecal samples of colorectal cancer and adenoma patients based on the proteome. Part A and B depict the outcomes of the Elastic Net (EN) and Least Absolute Shrinkage and Selection Operator (LASSO) feature selection methods, respectively. The light blue color in both methods indicates the first quartile of the ranked features across 100 iterations. Selected proteins were HP, CEACAM8, PRSS8, MUC2, CP, SERPINA3, SGSH and FCGBP. In part C, the relatedness of the selected markers is depicted using Principal Component Analysis (PCA). Part D depicts the stability plot obtained with logistic regression for the combined marker panel that has been selected. Corresponding area under the curve (AUC) is presented in blue.

Supplementary Figure 6. Machine learning pipeline amino acid profiles for colorectal cancer versus controls


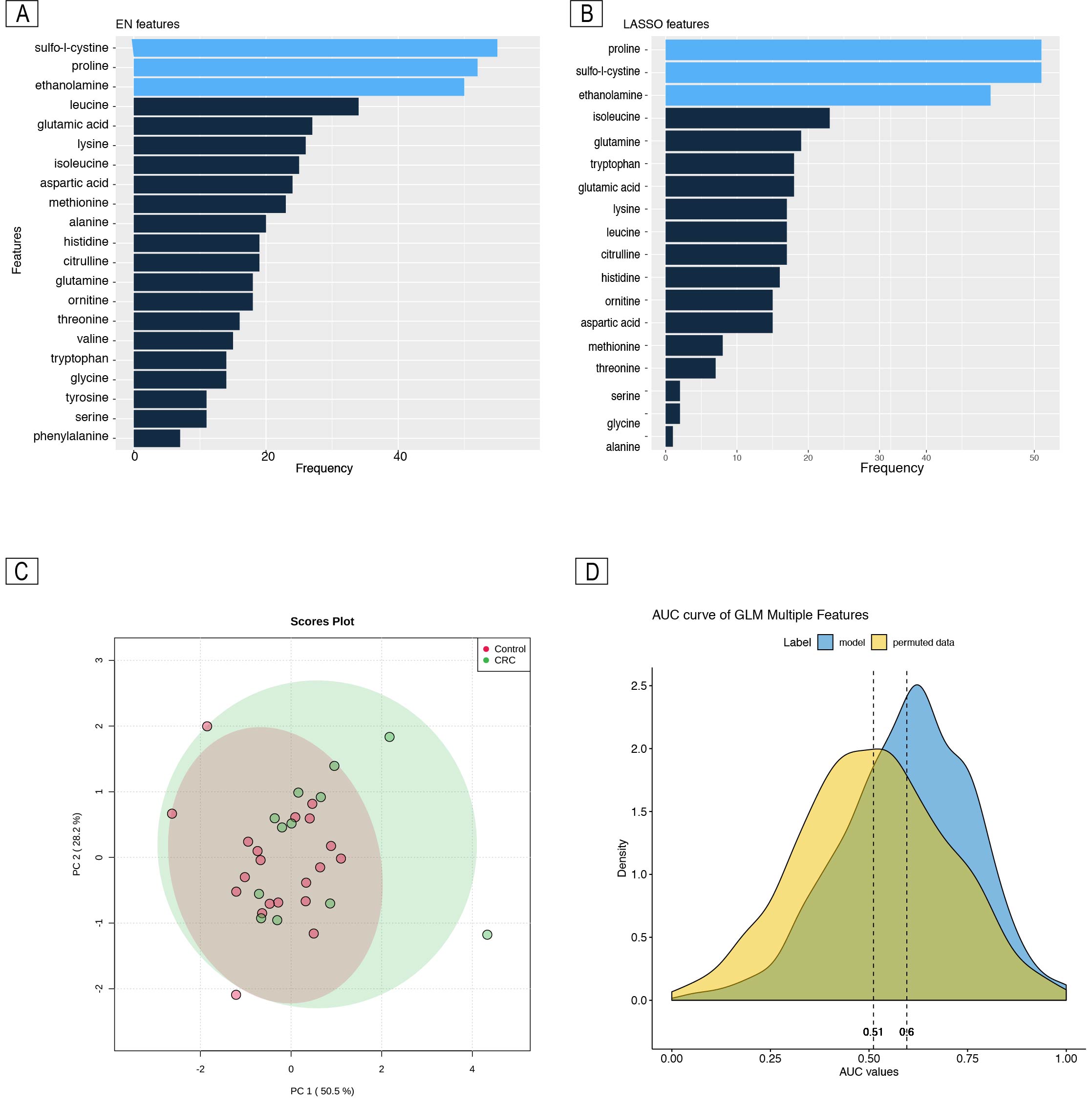


Supplementary Figure 6. The entire machine learning pipeline for the comparison between fecal samples of colorectal cancer patients and controls based on amino acids. Part A and B depict the outcomes of the Elastic Net (EN) and Least Absolute Shrinkage and Selection Operator (LASSO) feature selection methods, respectively. The light blue color in both methods indicates the first quartile of the ranked features across 100 iterations. In part C, the relatedness of the selected markers is depicted using Principal Component Analysis (PCA). Part D depicts the stability plot obtained with logistic regression for the combined marker panel that has been selected. Corresponding area under the curve (AUC) is presented in blue.


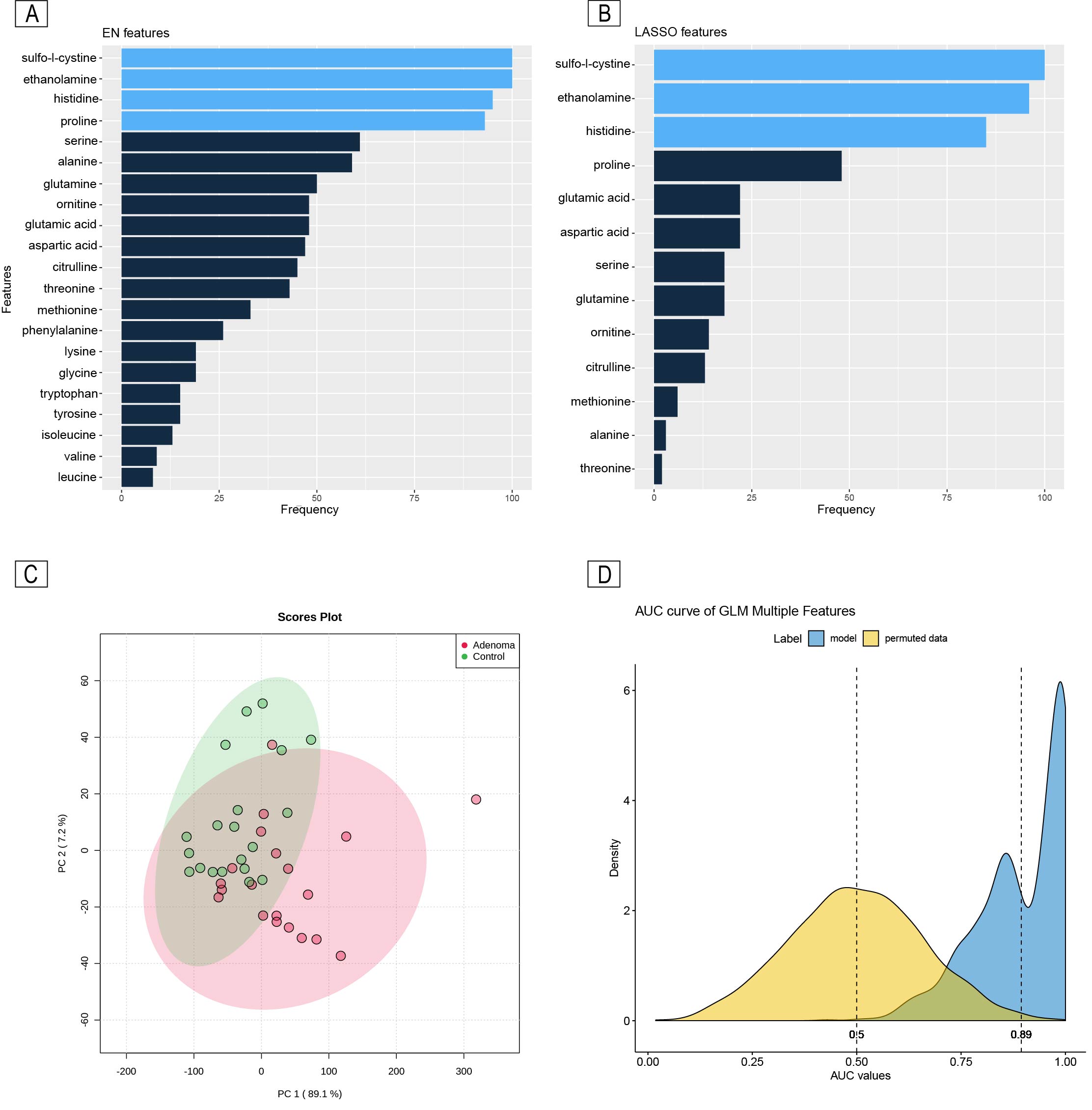
Supplementary Figure 7. Machine learning pipeline amino acid profiles for adenoma versus controls

Supplementary Figure 7. The entire machine learning pipeline for the comparison between fecal samples of adenoma patients and controls based on amino acids. Part A and B depict the outcomes of the Elastic Net (EN) and Least Absolute Shrinkage and Selection Operator (LASSO) feature selection methods, respectively. The light blue color in both methods indicates the first quartile of the ranked features across 100 iterations. In part C, the relatedness of the selected markers is depicted using Principal Component Analysis (PCA). Part D depicts the stability plot obtained with logistic regression for the combined marker panel that has been selected. Corresponding area under the curve (AUC) is presented in blue.


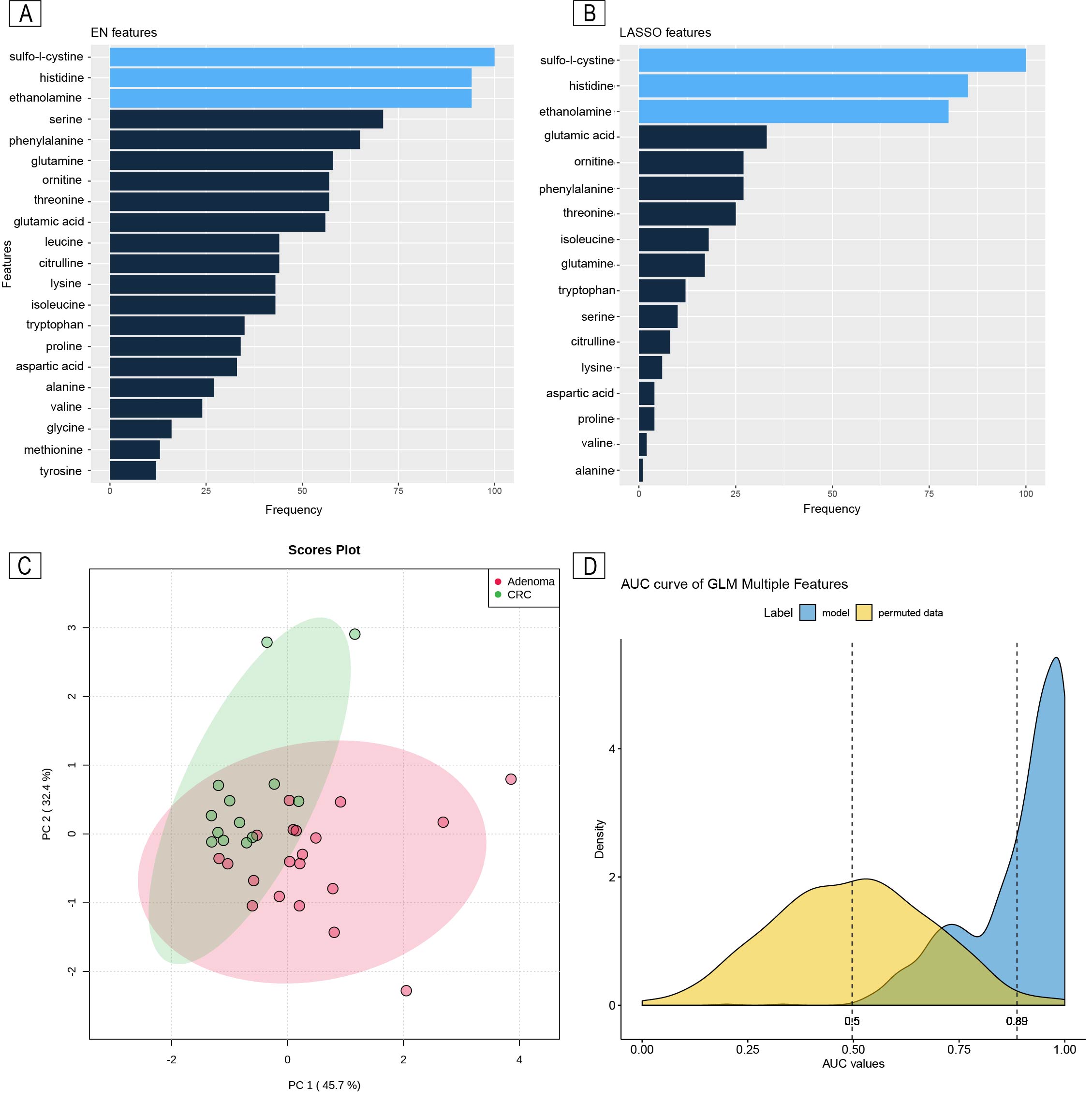
Supplementary Figure 8. Machine learning pipeline amino acids for colorectal cancer versus adenomas

Supplementary Figure 8. The entire machine learning pipeline for the comparison between fecal samples of colorectal cancer patients and adenomas based on amino acids. Part A and B depict the outcomes of the Elastic Net (EN) and Least Absolute Shrinkage and Selection Operator (LASSO) feature selection methods, respectively. The light blue color in both methods indicates the first quartile of the ranked features across 100 iterations. In part C, the relatedness of the selected markers is depicted using Principal Component Analysis (PCA). Part D depicts the stability plot obtained with logistic regression for the combined marker panel that has been selected. Corresponding area under the curve (AUC) is presented in blue.


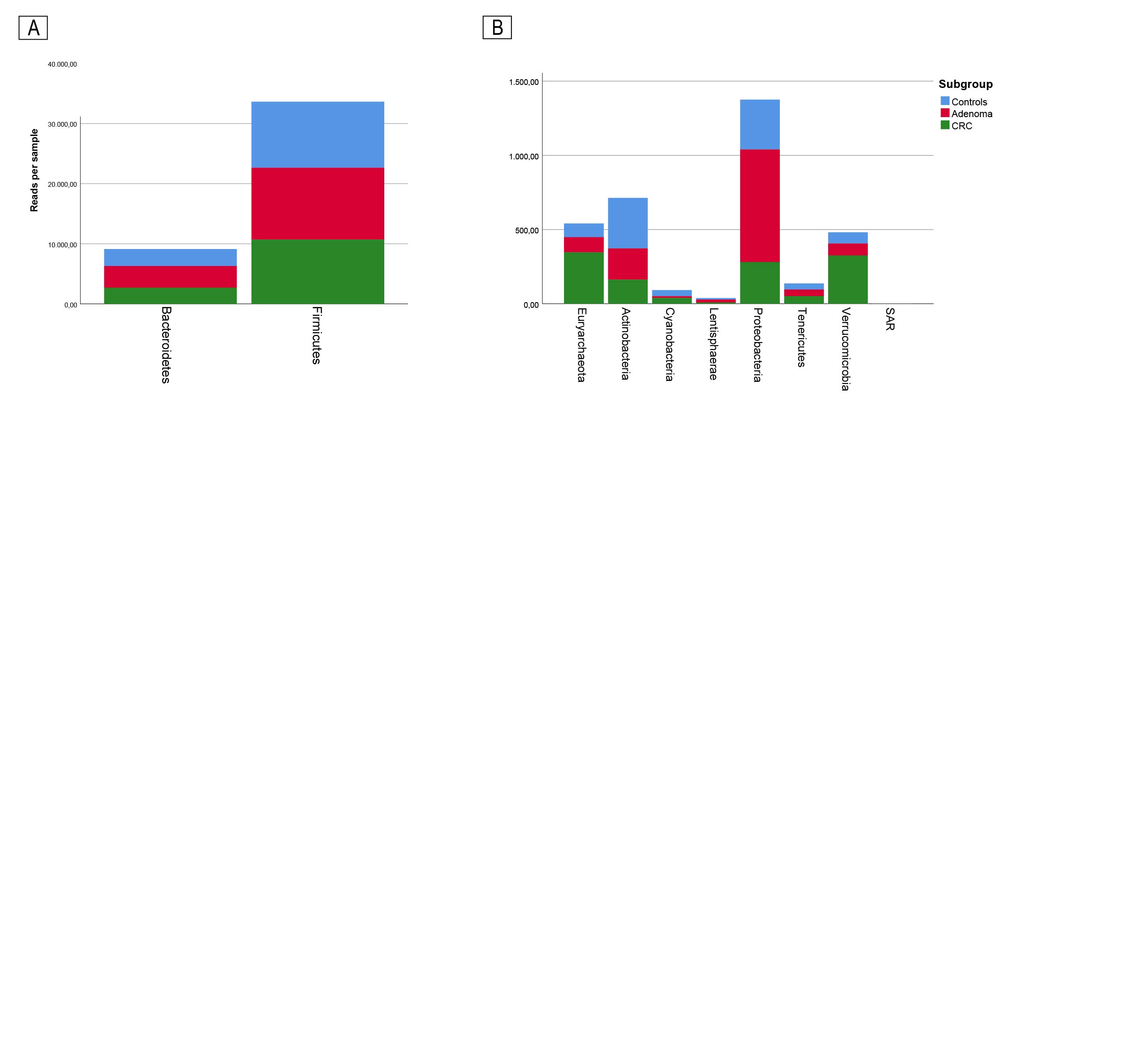
Supplementary Figure 9 Distribution of microbiota on Phylum level visualized in Bar Plots

Supplementary Figure 9. A: Bar plots for abundance of Phyla Bacteroidetes and Firmicutes across groups of colorectal cancer, adenomas and controls. B: Bar plots for the abundance of the Phyla Euryarchaeota, Actinobacteria, Cyanobacteria, Lentisphaerae, Proteobacteria, Tenericutes, Verrucomicrobia and SAR. The latter was, in this case, only represented by the Eukaryota *Blastocystis hominis.*


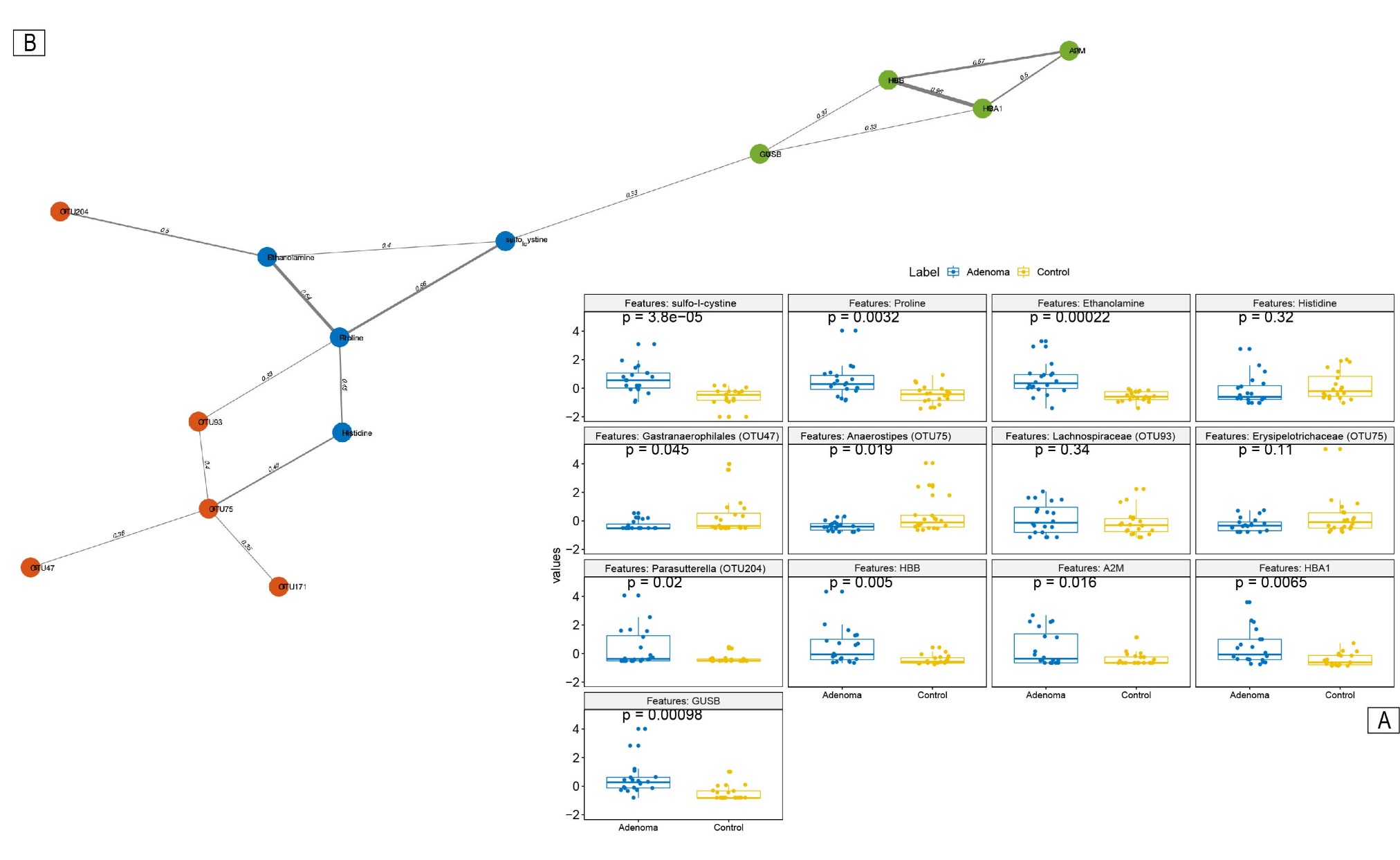
Supplementary Figure 10. Integration network for the comparison between adenomas and controls

Supplementary Figure 11. Adenoma versus control network. A: Differentially expressed features of proteins, bacterial taxa and amino acids data were selected using Least Absolute Shrinkage and Selection Operator (LASSO) and Elastic Net (EN). The significant correlations among the features are calculated at p<0.05.. B: Features in the boxplots correspond to the following markers (from left to right, above to below): sulfo-l-cystine, proline, ethanolamine, histidine, *Gastranaerophilales* (OTU47), *Anaerostipes* (OTU75), *Lachnospiraceae* (OTU93), *Erysipelotrichaceae* (OTU75), *Parasutterella* (OTU204), HBB, A2M, HBA1 and GUSB*.* B: Based on Pearson correlation, selected markers from these separate datasets were combined into one integration model. In this figure, solely correlations with a coefficient above 0.3 or below -0.3 have been depicted. Each type of marker is represented as node in different colors: Proteins as green, amino acids as blue and microbial taxa as red. The correlation values are used as edge in the nodes/features.


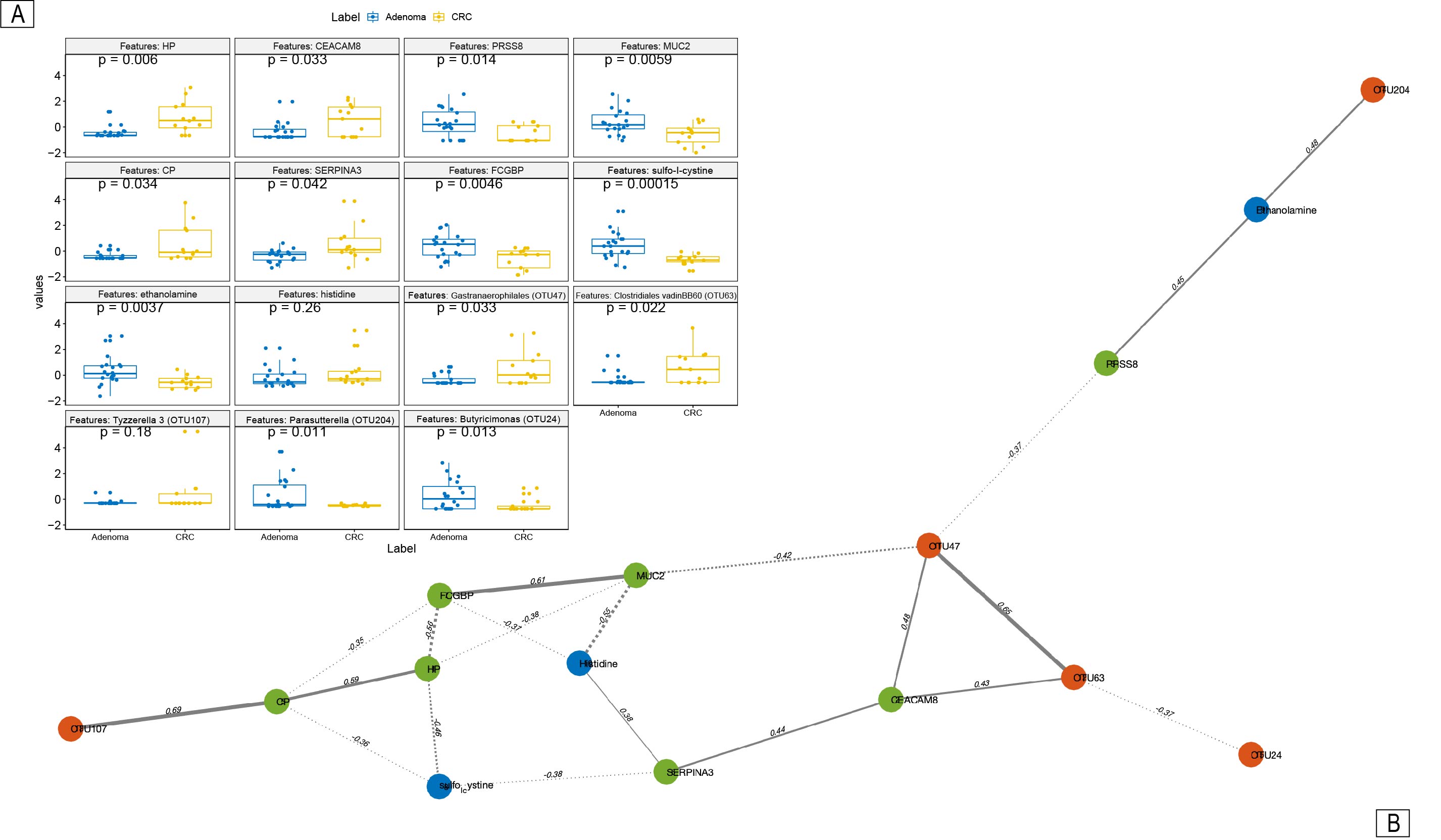
Supplementary Figure 11. Integration network for the comparison between colorectal cancer and adenomas

Supplementary Figure 12. Adenoma versus control network. A: Differentially expressed features of proteins, bacterial taxa and amino acids data were selected using Least Absolute Shrinkage and Selection Operator (LASSO) and Elastic Net (EN). The significant correlations among the features are calculated at p<0.05. B: Features in the boxplots correspond to the following markers (from left to right, above to below): HP, CEACAM8, PRSS8, MUC2, CP, SERPINA3, FCGBP, sulfo-l-cystine, ethanolamine, histidine, *Gastronaelophilales* (OTU47), *Clostridialis* (OTU63), *Tyzerrella 3* (OTU107)*, Parasutterella* (OTU204)*, Butyricimonas* (OTU24)*.* B: Based on Pearson correlation, selected markers from these separate datasets were combined into one integration model. In this figure, solely correlations with a coefficient above 0.3 or below -0.3 have been depicted. Each type of marker is represented as node in different colors: Proteins as green, amino acids as blue and microbial taxa as red. The correlation values are used as edge in the nodes/features. Abbreviations: CRC, colorectal cancer

Supplementary Figure 12. Integration network including all correlations per comparison

A. CRC versus control


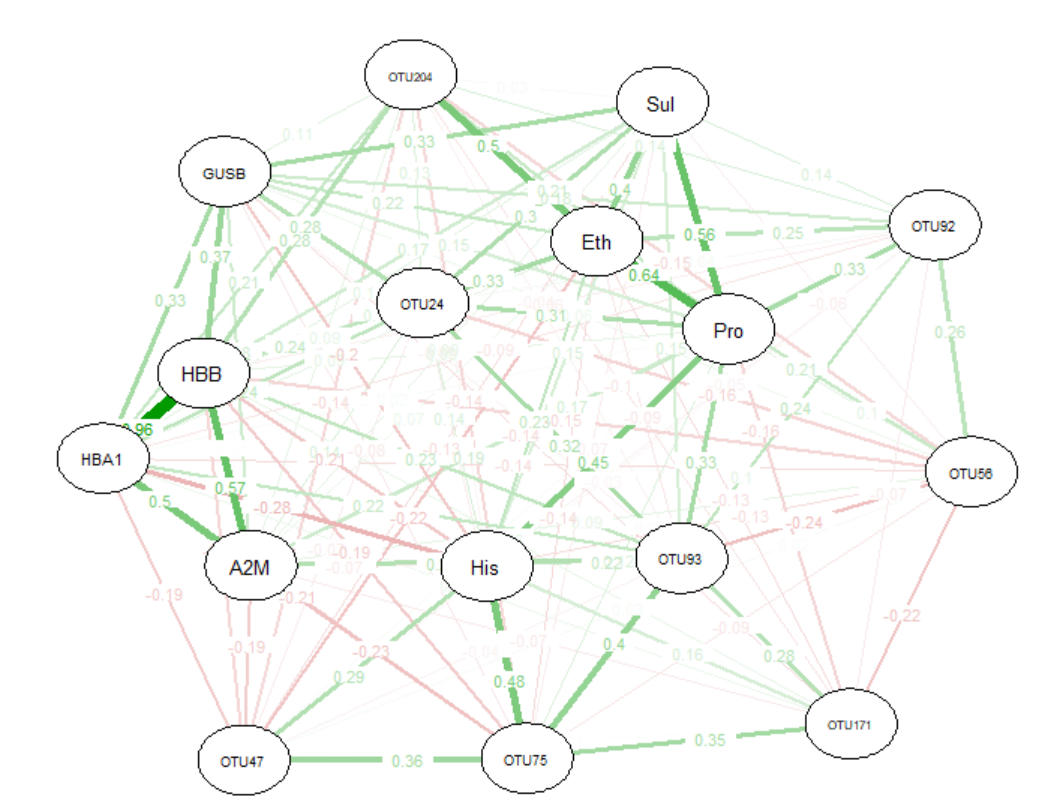


B. Adenoma versus control


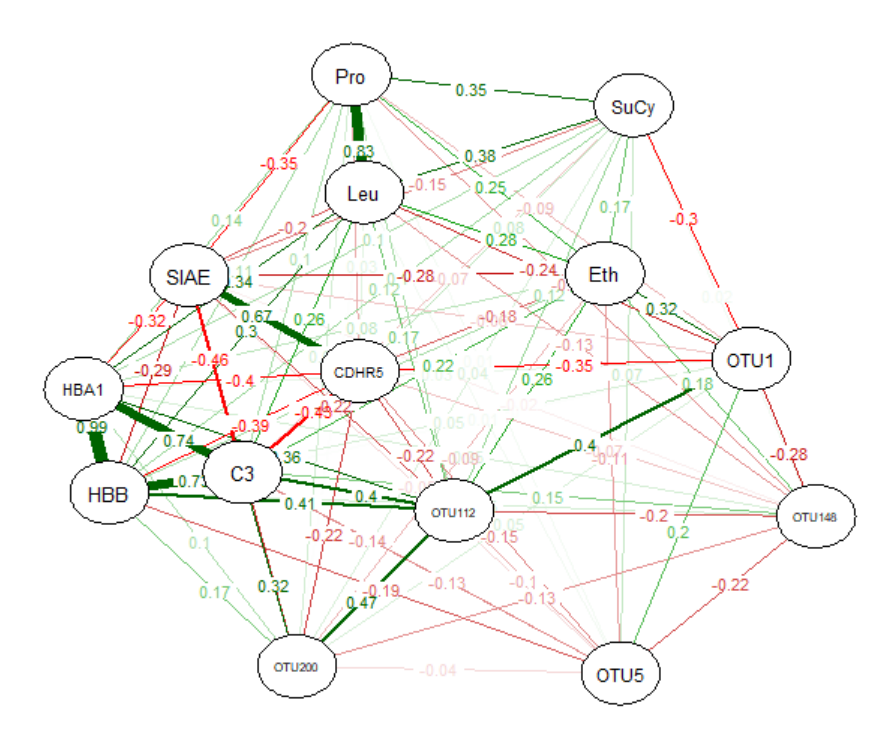

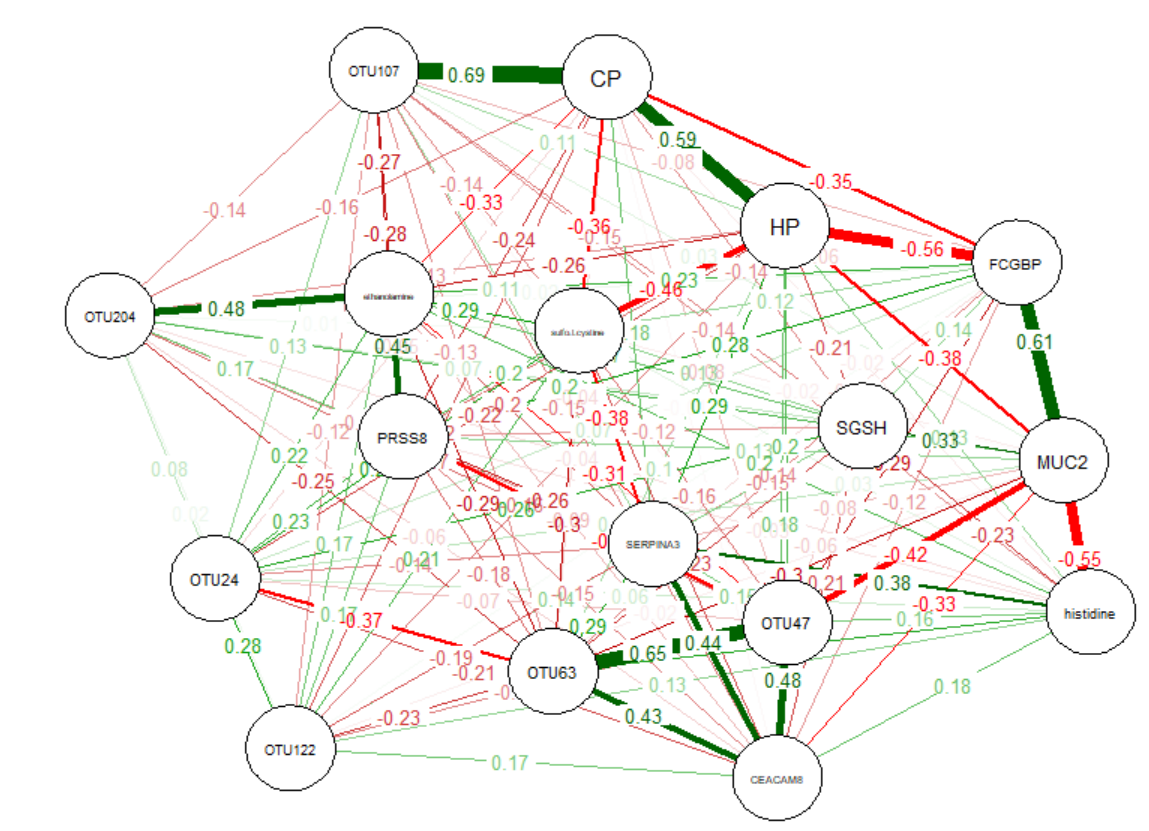


C. CRC versus adenoma

Supplementary Figure 13. Network clusters of the differentially expressed proteins, bacterial taxa and amino acids selected based on their contributions for outcome variables using Least Absolute Shrinkage and Selection Operator (LASSO) and Elastic Net (EN). A. Colorectal cancer versus controls, B. Adenoma versus controls, C. Colorectal cancer versus adenoma. All possible correlations are included in these network models. Green lines represent positive correlations, red lines represent negative correlations. Linewidth represents strength of correlations. Abbreviations: CRC, colorectal cancer.

Supplementary Table 1. Selected markers for differentiation between colorectal cancer and controls

| **Features** | **Description** | **Regulation in CRC** | **AUC** | **p-value** |
| --- | --- | --- | --- | --- |
| *Proteins* |  |  |  |  |
| C3 | Complement C3 | Upregulation | 0·752272727 | 0·000172133 |
| CDHR5 | Cadherin-related family member 5 | Downregulation | 0·865909091 | 0·000502243 |
| CP | Ceruloplasmin | Upregulation | 0·84038 | 0·0016791 |
| HBA1 | Haemoglobin subunit alpha | Upregulation | 0·954545455 | 0·000281262 |
| HBB | Haemoglobin subunit beta | Upregulation | 0·884090909 | 8·95E-05 |
| HP | Haptoglobin | Upregulation | 0·85577 | 2·42E-05 |
| SERPINA3 | Alpha-1-antichymotrypsin His-Pro-less | Upregulation | 0·75769 | 0·0085865 |
| SIAE | Sialate O-acetylesterase | Downregulation | 0·890909091 | 3·91E-05 |
|  |  |  |  |  |
| *Microbiota* |  |  |  |  |
| OTU1 | Methanobrevibacter | Similar | 0·5 | 0·809005015 |
| OTU5 | Bifidobacter | Downregulation | 0·772727273 | 0·085113461 |
| OTU112 | Eubacterium halii | Upregulation | 0·640909091 | 0·092573342 |
| OTU148 | Ruminococcaceae UCG-003 | Upregulation | 0·622727273 | 0·306207513 |
| OTU200 | Desulfovibrio | Upregulation | 0·688636364 | 0·09026708 |
|  |  |  |  |  |
| *Amino acids* |  |  |  |  |
| Ethanolamine | NA | Upregulation | 0·659090909 | 0·066834564 |
| Proline | NA | Upregulation | 0·718181818 | 0·054506197 |
| Sulfo-l-cystine | NA | Similar | 0·563636364 | 0·423089782 |

Supplementary Table 1. Markers were selected based on Least Absolute Shrinkage and Selection Operator (LASSO) and Elastic Net (EN) feature selection methods. A total of eight proteins, five bacterial taxa and three amino acids were selected to differentiate between patients with colorectal cancer and controls based on fecal samples. Abbreviations CRC, colorectal cancer; AUC, area under the curve

Supplementary Table 2. Selected markers for differentiation between adenoma and controls

| **Features** | **Description** | **Regulation in adenomas** | **AUC** | **p-value** |
| --- | --- | --- | --- | --- |
| *Proteins* |  |  |  |  |
| A2M | Alpha-2-macroglobulin | Upregulation | 0·6725 | 0·013756 |
| GUSB | Beta-glucuronidase | Upregulation | 0·87 | 0·001431 |
| HBA1 | Haemoglobin subunit alpha | Upregulation | 0·76 | 0·005133 |
| HBB | Haemoglobin subunit beta | Upregulation | 0·7875 | 0·003388 |
|  |  |  |  |  |
| *Microbiota* |  |  |  |  |
| OTU24 | *Butyricimonas* | Upregulation | 0·7775 | 0·002411 |
| OTU47 | *Gastranaerophilales* | Downregulation | 0·67625 | 0·04038 |
| OTU56 | *Streptococcus* | Similar | 0·5125 | 0·183189 |
| OTU75 | *Anaerostipes* | Downregulation | 0·7075 | 0·01565 |
| OTU92 | *Lachnospiraceae FCS020* | Upregulation | 0·65125 | 0·066349 |
| OTU93 | *Lachnospiraceae ND3007* | Upregulation | 0·57 | 0·333957 |
| OTU171 | *Erysipelotrichaceae UCG-003* | Downregulation | 0·61875 | 0·107381 |
| OTU204 | *Parasutterella* | Upregulation | 0·68875 | 0·015776 |
|  |  |  |  |  |
| *Amino acids* |  |  |  |  |
| Ethanolamine | NA | Upregulation | 0·8925 | 8·53E-05 |
| Histidine | NA | Downregulation | 0·6325 | 0·317336 |
| Proline | NA | Upregulation | 0·78375 | 0·002737 |
| Sulfo-l-cystine | NA | Upregulation | 0·87375 | 2·58E-05 |

Supplementary Table 2. Markers were selected based on Least Absolute Shrinkage and Selection Operator (LASSO) and Elastic Net (EN) feature selection methods. A total of four proteins, eight bacterial taxa and four amino acids were selected to differentiate between adenoma patients and controls based on fecal samples. Abbreviations AUC, area under the curve.

Supplementary Table 3 Selected markers for differentiation between colorectal cancer and adenoma

| **Features** |  |  | **Description** | **Regulation in CRC** | **AUC** | **p-value** |
| --- | --- | --- | --- | --- | --- | --- |
| *Proteins* |  |  |  |  |  |  |
| CEACAM8 |  |  | Carcinoembryonic antigen-related cell adhesion molecule 8 | Upregulation | 0·682692308 | 0·01138 |
| CP |  |  | Ceruloplasmin | Upregulation | 0·748076923 | 0·00684 |
| FCGBP |  |  | IgGFc-binding protein | Downregulation | 0·765384615 | 0·00582 |
| HP |  |  | Haptoglobin | Upregulation | 0·792307692 | 0·00056 |
| MUC2 |  |  | Mucin-2 | Downregulation | 0·75 | 0·00676 |
| PRSS8 |  |  | Prostasin | Downregulation | 0·705769231 | 0·02345 |
| SERPINA3 |  |  | Alpha-1-antichymotrypsin;Alpha-1-antichymotrypsin His-Pro-less | Upregulation | 0·753846154 | 0·01223 |
| SGSH |  |  | N-sulphoglucosamine sulphohydrolase | Downregulation | 0·751923077 | 0·02796 |
|  |  |  |  |  |  |  |
| *Microbiota* |  |  |  |  |  |  |
| OTU24 |  |  | Butyricimonas | Downregulation | 0·718673921 | 0·00487 |
| OTU47 |  |  | Gastranaerophilales | Upregulation | 0·730769231 | 0·00723 |
| OTU63 |  |  | Clostridiales vadinBB60 group | Upregulation | 0·732692308 | 0·00499 |
| OTU107 |  |  | Tyzzerella 3 | Upregulation | 0·615384615 | 0·0882 |
| OTU122 |  |  | Peptococcaceae | Downregulation | 0·675 | 0·06091 |
| OTU204 |  |  | Parasutterella | Downregulation | 0·690384615 | 0·03196 |
|  |  |  |  |  |  |  |
| *Amino acids* |  |  |  |  |  |  |
| Ethanolamine |  |  | NA | Downregulation | 0·801923077 | 0·01034 |
| Histidine |  |  | NA | Upregulation | 0·671153846 | 0·21333 |
| Sulfo-l-cystine |  |  | NA | Downregulation | 0·861538462 | 0·00074 |

Supplementary Table 3. Markers were selected based on Least Absolute Shrinkage and Selection Operator (LASSO) and Elastic Net (EN) feature selection methods. A total of eight proteins, six bacterial taxa and three amino acids were selected to differentiate between colorectal cancer and adenoma patients based on fecal samples. Abbreviations CRC, colorectal cancer; AUC, area under the curve.

Supplementary Table 4. Overview of operational taxonomic units

| **OTU** | **Kingdom** | **Phylum** | **Class** | **Order** | **Family** | **Genus** |
| --- | --- | --- | --- | --- | --- | --- |
| OTU1 | Archaea | Euryarchaeota | Methanobacteria | Methanobacteriales | Methanobacteriaceae | Methanobrevibacter |
| OTU2 | Archaea | Euryarchaeota | Methanobacteria | Methanobacteriales | Methanobacteriaceae | Methanosphaera |
| OTU3 | Archaea | Euryarchaeota | Thermoplasmata | Methanomassiliicoccales | Methanomassiliicoccaceae | Methanomassiliicoccus |
| OTU4 | Bacteria | Actinobacteria | Actinobacteria | Actinomycetales | Actinomycetaceae | Actinomyces |
| OTU5 | Bacteria | Actinobacteria | Actinobacteria | Bifidobacteriales | Bifidobacteriaceae | Bifidobacterium |
| OTU6 | Bacteria | Actinobacteria | Actinobacteria | Bifidobacteriales | Bifidobacteriaceae |  |
| OTU7 | Bacteria | Actinobacteria | Coriobacteriia | Coriobacteriales | Atopobiaceae | Atopobium |
| OTU8 | Bacteria | Actinobacteria | Coriobacteriia | Coriobacteriales | Atopobiaceae | Libanicoccus |
| OTU9 | Bacteria | Actinobacteria | Coriobacteriia | Coriobacteriales | Atopobiaceae | Olsenella |
| OTU10 | Bacteria | Actinobacteria | Coriobacteriia | Coriobacteriales | Coriobacteriaceae | Collinsella |
| OTU11 | Bacteria | Actinobacteria | Coriobacteriia | Coriobacteriales | Coriobacteriales Incertae Sedis | uncultured |
| OTU12 | Bacteria | Actinobacteria | Coriobacteriia | Coriobacteriales | Eggerthellaceae | Adlercreutzia |
| OTU13 | Bacteria | Actinobacteria | Coriobacteriia | Coriobacteriales | Eggerthellaceae | Eggerthella |
| OTU14 | Bacteria | Actinobacteria | Coriobacteriia | Coriobacteriales | Eggerthellaceae | Enterorhabdus |
| OTU15 | Bacteria | Actinobacteria | Coriobacteriia | Coriobacteriales | Eggerthellaceae | Senegalimassilia |
| OTU16 | Bacteria | Actinobacteria | Coriobacteriia | Coriobacteriales | Eggerthellaceae | Slackia |
| OTU17 | Bacteria | Actinobacteria | Coriobacteriia | Coriobacteriales | Eggerthellaceae | uncultured |
| OTU18 | Bacteria | Actinobacteria | Coriobacteriia | Coriobacteriales | Eggerthellaceae |  |
| OTU19 | Bacteria | Actinobacteria | Coriobacteriia | Coriobacteriales | uncultured | uncultured bacterium |
| OTU20 | Bacteria | Bacteroidetes | Bacteroidia | Bacteroidales | Bacteroidaceae | Bacteroides |
| OTU21 | Bacteria | Bacteroidetes | Bacteroidia | Bacteroidales | Barnesiellaceae | Barnesiella |
| OTU22 | Bacteria | Bacteroidetes | Bacteroidia | Bacteroidales | Barnesiellaceae | Coprobacter |
| OTU23 | Bacteria | Bacteroidetes | Bacteroidia | Bacteroidales | Barnesiellaceae | uncultured |
| OTU24 | Bacteria | Bacteroidetes | Bacteroidia | Bacteroidales | Marinifilaceae | Butyricimonas |
| OTU25 | Bacteria | Bacteroidetes | Bacteroidia | Bacteroidales | Marinifilaceae | Odoribacter |
| OTU26 | Bacteria | Bacteroidetes | Bacteroidia | Bacteroidales | Marinifilaceae | Sanguibacteroides |
| OTU27 | Bacteria | Bacteroidetes | Bacteroidia | Bacteroidales | Muribaculaceae | uncultured Porphyromonadaceae bacterium |
| OTU28 | Bacteria | Bacteroidetes | Bacteroidia | Bacteroidales | Muribaculaceae | uncultured bacterium |
| OTU29 | Bacteria | Bacteroidetes | Bacteroidia | Bacteroidales | Muribaculaceae | uncultured organism |
| OTU30 | Bacteria | Bacteroidetes | Bacteroidia | Bacteroidales | Muribaculaceae |  |
| OTU31 | Bacteria | Bacteroidetes | Bacteroidia | Bacteroidales | Porphyromonadaceae | Porphyromonas |
| OTU32 | Bacteria | Bacteroidetes | Bacteroidia | Bacteroidales | Prevotellaceae | Alloprevotella |
| OTU33 | Bacteria | Bacteroidetes | Bacteroidia | Bacteroidales | Prevotellaceae | Paraprevotella |
| OTU34 | Bacteria | Bacteroidetes | Bacteroidia | Bacteroidales | Prevotellaceae | Prevotella |
| OTU35 | Bacteria | Bacteroidetes | Bacteroidia | Bacteroidales | Prevotellaceae | Prevotella 2 |
| OTU36 | Bacteria | Bacteroidetes | Bacteroidia | Bacteroidales | Prevotellaceae | Prevotella 7 |
| OTU37 | Bacteria | Bacteroidetes | Bacteroidia | Bacteroidales | Prevotellaceae | Prevotella 9 |
| OTU38 | Bacteria | Bacteroidetes | Bacteroidia | Bacteroidales | Prevotellaceae | Prevotellaceae NK3B31 group |
| OTU39 | Bacteria | Bacteroidetes | Bacteroidia | Bacteroidales | Prevotellaceae | Prevotellaceae UCG-001 |
| OTU40 | Bacteria | Bacteroidetes | Bacteroidia | Bacteroidales | Prevotellaceae | uncultured |
| OTU41 | Bacteria | Bacteroidetes | Bacteroidia | Bacteroidales | Rikenellaceae | Alistipes |
| OTU42 | Bacteria | Bacteroidetes | Bacteroidia | Bacteroidales | Rikenellaceae | Rikenellaceae RC9 gut group |
| OTU43 | Bacteria | Bacteroidetes | Bacteroidia | Bacteroidales | Tannerellaceae | Parabacteroides |
| OTU44 | Bacteria | Bacteroidetes | Bacteroidia | Bacteroidales | uncultured | gut metagenome |
| OTU45 | Bacteria | Bacteroidetes | Bacteroidia | Bacteroidales | uncultured | uncultured bacterium |
| OTU46 | Bacteria | Bacteroidetes | Bacteroidia | Flavobacteriales | Flavobacteriaceae | uncultured |
| OTU47 | Bacteria | Cyanobacteria | Melainabacteria | Gastranaerophilales | uncultured bacterium | uncultured bacterium |
| OTU48 | Bacteria | Cyanobacteria | Melainabacteria | Gastranaerophilales | uncultured rumen bacterium | uncultured rumen bacterium |
| OTU49 | Bacteria | Cyanobacteria | Oxyphotobacteria | Chloroplast |  |  |
| OTU50 | Bacteria | Firmicutes | Bacilli | Bacillales | Family XI | Gemella |
| OTU51 | Bacteria | Firmicutes | Bacilli | Lactobacillales | Enterococcaceae | Enterococcus |
| OTU52 | Bacteria | Firmicutes | Bacilli | Lactobacillales | Lactobacillaceae | Lactobacillus |
| OTU53 | Bacteria | Firmicutes | Bacilli | Lactobacillales | Leuconostocaceae | Leuconostoc |
| OTU54 | Bacteria | Firmicutes | Bacilli | Lactobacillales | Leuconostocaceae | Weissella |
| OTU55 | Bacteria | Firmicutes | Bacilli | Lactobacillales | Streptococcaceae | Lactococcus |
| OTU56 | Bacteria | Firmicutes | Bacilli | Lactobacillales | Streptococcaceae | Streptococcus |
| OTU57 | Bacteria | Firmicutes | Clostridia | Clostridiales | Christensenellaceae | Christensenellaceae R-7 group |
| OTU58 | Bacteria | Firmicutes | Clostridia | Clostridiales | Christensenellaceae | uncultured |
| OTU59 | Bacteria | Firmicutes | Clostridia | Clostridiales | Christensenellaceae |  |
| OTU60 | Bacteria | Firmicutes | Clostridia | Clostridiales | Clostridiaceae 1 | Clostridium sensu stricto 1 |
| OTU61 | Bacteria | Firmicutes | Clostridia | Clostridiales | Clostridiales vadinBB60 group | uncultured bacterium |
| OTU62 | Bacteria | Firmicutes | Clostridia | Clostridiales | Clostridiales vadinBB60 group | uncultured organism |
| OTU63 | Bacteria | Firmicutes | Clostridia | Clostridiales | Clostridiales vadinBB60 group |  |
| OTU64 | Bacteria | Firmicutes | Clostridia | Clostridiales | Defluviitaleaceae | Defluviitaleaceae UCG-011 |
| OTU65 | Bacteria | Firmicutes | Clostridia | Clostridiales | Eubacteriaceae | Anaerofustis |
| OTU66 | Bacteria | Firmicutes | Clostridia | Clostridiales | Eubacteriaceae | Eubacterium |
| OTU67 | Bacteria | Firmicutes | Clostridia | Clostridiales | Family XI | Parvimonas |
| OTU68 | Bacteria | Firmicutes | Clostridia | Clostridiales | Family XIII | Family XIII AD3011 group |
| OTU69 | Bacteria | Firmicutes | Clostridia | Clostridiales | Family XIII | Family XIII UCG-001 |
| OTU70 | Bacteria | Firmicutes | Clostridia | Clostridiales | Family XIII | Mogibacterium |
| OTU71 | Bacteria | Firmicutes | Clostridia | Clostridiales | Family XIII | [Eubacterium] brachy group |
| OTU72 | Bacteria | Firmicutes | Clostridia | Clostridiales | Family XIII | [Eubacterium] nodatum group |
| OTU73 | Bacteria | Firmicutes | Clostridia | Clostridiales | Family XIII |  |
| OTU74 | Bacteria | Firmicutes | Clostridia | Clostridiales | Lachnospiraceae | Agathobacter |
| OTU75 | Bacteria | Firmicutes | Clostridia | Clostridiales | Lachnospiraceae | Anaerostipes |
| OTU76 | Bacteria | Firmicutes | Clostridia | Clostridiales | Lachnospiraceae | Blautia |
| OTU77 | Bacteria | Firmicutes | Clostridia | Clostridiales | Lachnospiraceae | Butyrivibrio |
| OTU78 | Bacteria | Firmicutes | Clostridia | Clostridiales | Lachnospiraceae | CAG-56 |
| OTU79 | Bacteria | Firmicutes | Clostridia | Clostridiales | Lachnospiraceae | Coprococcus 1 |
| OTU80 | Bacteria | Firmicutes | Clostridia | Clostridiales | Lachnospiraceae | Coprococcus 2 |
| OTU81 | Bacteria | Firmicutes | Clostridia | Clostridiales | Lachnospiraceae | Coprococcus 3 |
| OTU82 | Bacteria | Firmicutes | Clostridia | Clostridiales | Lachnospiraceae | Dorea |
| OTU83 | Bacteria | Firmicutes | Clostridia | Clostridiales | Lachnospiraceae | Eisenbergiella |
| OTU84 | Bacteria | Firmicutes | Clostridia | Clostridiales | Lachnospiraceae | Epulopiscium |
| OTU85 | Bacteria | Firmicutes | Clostridia | Clostridiales | Lachnospiraceae | Fusicatenibacter |
| OTU86 | Bacteria | Firmicutes | Clostridia | Clostridiales | Lachnospiraceae | GCA-900066575 |
| OTU87 | Bacteria | Firmicutes | Clostridia | Clostridiales | Lachnospiraceae | Howardella |
| OTU88 | Bacteria | Firmicutes | Clostridia | Clostridiales | Lachnospiraceae | Hungatella |
| OTU89 | Bacteria | Firmicutes | Clostridia | Clostridiales | Lachnospiraceae | Lachnoclostridium |
| OTU90 | Bacteria | Firmicutes | Clostridia | Clostridiales | Lachnospiraceae | Lachnospira |
| OTU91 | Bacteria | Firmicutes | Clostridia | Clostridiales | Lachnospiraceae | Lachnospiraceae AC2044 group |
| OTU92 | Bacteria | Firmicutes | Clostridia | Clostridiales | Lachnospiraceae | Lachnospiraceae FCS020 group |
| OTU93 | Bacteria | Firmicutes | Clostridia | Clostridiales | Lachnospiraceae | Lachnospiraceae ND3007 group |
| OTU94 | Bacteria | Firmicutes | Clostridia | Clostridiales | Lachnospiraceae | Lachnospiraceae NK4A136 group |
| OTU95 | Bacteria | Firmicutes | Clostridia | Clostridiales | Lachnospiraceae | Lachnospiraceae NK4B4 group |
| OTU96 | Bacteria | Firmicutes | Clostridia | Clostridiales | Lachnospiraceae | Lachnospiraceae UCG-001 |
| OTU97 | Bacteria | Firmicutes | Clostridia | Clostridiales | Lachnospiraceae | Lachnospiraceae UCG-003 |
| OTU98 | Bacteria | Firmicutes | Clostridia | Clostridiales | Lachnospiraceae | Lachnospiraceae UCG-004 |
| OTU99 | Bacteria | Firmicutes | Clostridia | Clostridiales | Lachnospiraceae | Lachnospiraceae UCG-010 |
| OTU100 | Bacteria | Firmicutes | Clostridia | Clostridiales | Lachnospiraceae | Marvinbryantia |
| OTU101 | Bacteria | Firmicutes | Clostridia | Clostridiales | Lachnospiraceae | Oribacterium |
| OTU102 | Bacteria | Firmicutes | Clostridia | Clostridiales | Lachnospiraceae | Roseburia |
| OTU103 | Bacteria | Firmicutes | Clostridia | Clostridiales | Lachnospiraceae | Sellimonas |
| OTU104 | Bacteria | Firmicutes | Clostridia | Clostridiales | Lachnospiraceae | Shuttleworthia |
| OTU105 | Bacteria | Firmicutes | Clostridia | Clostridiales | Lachnospiraceae | Syntrophococcus |
| OTU106 | Bacteria | Firmicutes | Clostridia | Clostridiales | Lachnospiraceae | Tyzzerella |
| OTU107 | Bacteria | Firmicutes | Clostridia | Clostridiales | Lachnospiraceae | Tyzzerella 3 |
| OTU108 | Bacteria | Firmicutes | Clostridia | Clostridiales | Lachnospiraceae | Tyzzerella 4 |
| OTU109 | Bacteria | Firmicutes | Clostridia | Clostridiales | Lachnospiraceae | [Bacteroides] pectinophilus group |
| OTU110 | Bacteria | Firmicutes | Clostridia | Clostridiales | Lachnospiraceae | [Eubacterium] eligens group |
| OTU111 | Bacteria | Firmicutes | Clostridia | Clostridiales | Lachnospiraceae | [Eubacterium] fissicatena group |
| OTU112 | Bacteria | Firmicutes | Clostridia | Clostridiales | Lachnospiraceae | [Eubacterium] hallii group |
| OTU113 | Bacteria | Firmicutes | Clostridia | Clostridiales | Lachnospiraceae | [Eubacterium] ruminantium group |
| OTU114 | Bacteria | Firmicutes | Clostridia | Clostridiales | Lachnospiraceae | [Eubacterium] ventriosum group |
| OTU115 | Bacteria | Firmicutes | Clostridia | Clostridiales | Lachnospiraceae | [Eubacterium] xylanophilum group |
| OTU116 | Bacteria | Firmicutes | Clostridia | Clostridiales | Lachnospiraceae | [Ruminococcus] gauvreauii group |
| OTU117 | Bacteria | Firmicutes | Clostridia | Clostridiales | Lachnospiraceae | [Ruminococcus] gnavus group |
| OTU118 | Bacteria | Firmicutes | Clostridia | Clostridiales | Lachnospiraceae | [Ruminococcus] torques group |
| OTU119 | Bacteria | Firmicutes | Clostridia | Clostridiales | Lachnospiraceae | uncultured |
| OTU120 | Bacteria | Firmicutes | Clostridia | Clostridiales | Lachnospiraceae |  |
| OTU121 | Bacteria | Firmicutes | Clostridia | Clostridiales | Peptococcaceae | Peptococcus |
| OTU122 | Bacteria | Firmicutes | Clostridia | Clostridiales | Peptococcaceae | uncultured |
| OTU123 | Bacteria | Firmicutes | Clostridia | Clostridiales | Peptostreptococcaceae | Intestinibacter |
| OTU124 | Bacteria | Firmicutes | Clostridia | Clostridiales | Peptostreptococcaceae | Peptostreptococcus |
| OTU125 | Bacteria | Firmicutes | Clostridia | Clostridiales | Peptostreptococcaceae | Romboutsia |
| OTU126 | Bacteria | Firmicutes | Clostridia | Clostridiales | Peptostreptococcaceae | Terrisporobacter |
| OTU127 | Bacteria | Firmicutes | Clostridia | Clostridiales | Ruminococcaceae | Anaerofilum |
| OTU128 | Bacteria | Firmicutes | Clostridia | Clostridiales | Ruminococcaceae | Anaerotruncus |
| OTU129 | Bacteria | Firmicutes | Clostridia | Clostridiales | Ruminococcaceae | Butyricicoccus |
| OTU130 | Bacteria | Firmicutes | Clostridia | Clostridiales | Ruminococcaceae | CAG-352 |
| OTU131 | Bacteria | Firmicutes | Clostridia | Clostridiales | Ruminococcaceae | Candidatus Soleaferrea |
| OTU132 | Bacteria | Firmicutes | Clostridia | Clostridiales | Ruminococcaceae | Caproiciproducens |
| OTU133 | Bacteria | Firmicutes | Clostridia | Clostridiales | Ruminococcaceae | DTU089 |
| OTU134 | Bacteria | Firmicutes | Clostridia | Clostridiales | Ruminococcaceae | Fecalibacterium |
| OTU135 | Bacteria | Firmicutes | Clostridia | Clostridiales | Ruminococcaceae | Flavonifractor |
| OTU136 | Bacteria | Firmicutes | Clostridia | Clostridiales | Ruminococcaceae | Fournierella |
| OTU137 | Bacteria | Firmicutes | Clostridia | Clostridiales | Ruminococcaceae | GCA-900066225 |
| OTU138 | Bacteria | Firmicutes | Clostridia | Clostridiales | Ruminococcaceae | Hydrogenoanaerobacterium |
| OTU139 | Bacteria | Firmicutes | Clostridia | Clostridiales | Ruminococcaceae | Negativibacillus |
| OTU140 | Bacteria | Firmicutes | Clostridia | Clostridiales | Ruminococcaceae | Oscillibacter |
| OTU141 | Bacteria | Firmicutes | Clostridia | Clostridiales | Ruminococcaceae | Oscillospira |
| OTU142 | Bacteria | Firmicutes | Clostridia | Clostridiales | Ruminococcaceae | Ruminiclostridium 1 |
| OTU143 | Bacteria | Firmicutes | Clostridia | Clostridiales | Ruminococcaceae | Ruminiclostridium 5 |
| OTU144 | Bacteria | Firmicutes | Clostridia | Clostridiales | Ruminococcaceae | Ruminiclostridium 6 |
| OTU145 | Bacteria | Firmicutes | Clostridia | Clostridiales | Ruminococcaceae | Ruminiclostridium 9 |
| OTU146 | Bacteria | Firmicutes | Clostridia | Clostridiales | Ruminococcaceae | Ruminococcaceae NK4A214 group |
| OTU147 | Bacteria | Firmicutes | Clostridia | Clostridiales | Ruminococcaceae | Ruminococcaceae UCG-002 |
| OTU148 | Bacteria | Firmicutes | Clostridia | Clostridiales | Ruminococcaceae | Ruminococcaceae UCG-003 |
| OTU149 | Bacteria | Firmicutes | Clostridia | Clostridiales | Ruminococcaceae | Ruminococcaceae UCG-004 |
| OTU150 | Bacteria | Firmicutes | Clostridia | Clostridiales | Ruminococcaceae | Ruminococcaceae UCG-005 |
| OTU151 | Bacteria | Firmicutes | Clostridia | Clostridiales | Ruminococcaceae | Ruminococcaceae UCG-008 |
| OTU152 | Bacteria | Firmicutes | Clostridia | Clostridiales | Ruminococcaceae | Ruminococcaceae UCG-009 |
| OTU153 | Bacteria | Firmicutes | Clostridia | Clostridiales | Ruminococcaceae | Ruminococcaceae UCG-010 |
| OTU154 | Bacteria | Firmicutes | Clostridia | Clostridiales | Ruminococcaceae | Ruminococcaceae UCG-013 |
| OTU155 | Bacteria | Firmicutes | Clostridia | Clostridiales | Ruminococcaceae | Ruminococcaceae UCG-014 |
| OTU156 | Bacteria | Firmicutes | Clostridia | Clostridiales | Ruminococcaceae | Ruminococcus 1 |
| OTU157 | Bacteria | Firmicutes | Clostridia | Clostridiales | Ruminococcaceae | Ruminococcus 2 |
| OTU158 | Bacteria | Firmicutes | Clostridia | Clostridiales | Ruminococcaceae | Subdoligranulum |
| OTU159 | Bacteria | Firmicutes | Clostridia | Clostridiales | Ruminococcaceae | UBA1819 |
| OTU160 | Bacteria | Firmicutes | Clostridia | Clostridiales | Ruminococcaceae | [Eubacterium] coprostanoligenes group |
| OTU161 | Bacteria | Firmicutes | Clostridia | Clostridiales | Ruminococcaceae | uncultured |
| OTU162 | Bacteria | Firmicutes | Clostridia | Clostridiales | Ruminococcaceae |  |
| OTU163 | Bacteria | Firmicutes | Clostridia | Clostridiales |  |  |
| OTU164 | Bacteria | Firmicutes | Clostridia | DTU014 | uncultured bacterium | uncultured bacterium |
| OTU165 | Bacteria | Firmicutes | Erysipelotrichia | Erysipelotrichales | Erysipelotrichaceae | Asteroleplasma |
| OTU166 | Bacteria | Firmicutes | Erysipelotrichia | Erysipelotrichales | Erysipelotrichaceae | Candidatus Stoquefichus |
| OTU167 | Bacteria | Firmicutes | Erysipelotrichia | Erysipelotrichales | Erysipelotrichaceae | Catenibacterium |
| OTU168 | Bacteria | Firmicutes | Erysipelotrichia | Erysipelotrichales | Erysipelotrichaceae | Catenisphaera |
| OTU169 | Bacteria | Firmicutes | Erysipelotrichia | Erysipelotrichales | Erysipelotrichaceae | Dielma |
| OTU170 | Bacteria | Firmicutes | Erysipelotrichia | Erysipelotrichales | Erysipelotrichaceae | Erysipelatoclostridium |
| OTU171 | Bacteria | Firmicutes | Erysipelotrichia | Erysipelotrichales | Erysipelotrichaceae | Erysipelotrichaceae UCG-003 |
| OTU172 | Bacteria | Firmicutes | Erysipelotrichia | Erysipelotrichales | Erysipelotrichaceae | Erysipelotrichaceae UCG-004 |
| OTU173 | Bacteria | Firmicutes | Erysipelotrichia | Erysipelotrichales | Erysipelotrichaceae | Erysipelotrichaceae UCG-006 |
| OTU174 | Bacteria | Firmicutes | Erysipelotrichia | Erysipelotrichales | Erysipelotrichaceae | Fecalitalea |
| OTU175 | Bacteria | Firmicutes | Erysipelotrichia | Erysipelotrichales | Erysipelotrichaceae | Holdemanella |
| OTU176 | Bacteria | Firmicutes | Erysipelotrichia | Erysipelotrichales | Erysipelotrichaceae | Holdemania |
| OTU177 | Bacteria | Firmicutes | Erysipelotrichia | Erysipelotrichales | Erysipelotrichaceae | Merdibacter |
| OTU178 | Bacteria | Firmicutes | Erysipelotrichia | Erysipelotrichales | Erysipelotrichaceae | Solobacterium |
| OTU179 | Bacteria | Firmicutes | Erysipelotrichia | Erysipelotrichales | Erysipelotrichaceae | Turicibacter |
| OTU180 | Bacteria | Firmicutes | Erysipelotrichia | Erysipelotrichales | Erysipelotrichaceae | [Clostridium] innocuum group |
| OTU181 | Bacteria | Firmicutes | Erysipelotrichia | Erysipelotrichales | Erysipelotrichaceae | uncultured |
| OTU182 | Bacteria | Firmicutes | Erysipelotrichia | Erysipelotrichales | Erysipelotrichaceae |  |
| OTU183 | Bacteria | Firmicutes | Negativicutes | Selenomonadales | Acidaminococcaceae | Acidaminococcus |
| OTU184 | Bacteria | Firmicutes | Negativicutes | Selenomonadales | Acidaminococcaceae | Phascolarctobacterium |
| OTU185 | Bacteria | Firmicutes | Negativicutes | Selenomonadales | Acidaminococcaceae | Succiniclasticum |
| OTU186 | Bacteria | Firmicutes | Negativicutes | Selenomonadales | Veillonellaceae | Allisonella |
| OTU187 | Bacteria | Firmicutes | Negativicutes | Selenomonadales | Veillonellaceae | Dialister |
| OTU188 | Bacteria | Firmicutes | Negativicutes | Selenomonadales | Veillonellaceae | Megamonas |
| OTU189 | Bacteria | Firmicutes | Negativicutes | Selenomonadales | Veillonellaceae | Megasphaera |
| OTU190 | Bacteria | Firmicutes | Negativicutes | Selenomonadales | Veillonellaceae | Mitsuokella |
| OTU191 | Bacteria | Firmicutes | Negativicutes | Selenomonadales | Veillonellaceae | Veillonella |
| OTU192 | Bacteria | Lentisphaerae | Lentisphaeria | Victivallales | Victivallaceae | Victivallis |
| OTU193 | Bacteria | Lentisphaerae | Lentisphaeria | Victivallales | Victivallaceae | uncultured bacterium |
| OTU194 | Bacteria | Lentisphaerae | Lentisphaeria | Victivallales | vadinBE97 | uncultured bacterium |
| OTU195 | Bacteria | Lentisphaerae | Lentisphaeria | Victivallales | vadinBE97 | uncultured rumen bacterium |
| OTU196 | Bacteria | Proteobacteria | Alphaproteobacteria | Rhodospirillales | uncultured | gut metagenome |
| OTU197 | Bacteria | Proteobacteria | Alphaproteobacteria | Rhodospirillales | uncultured | uncultured bacterium |
| OTU198 | Bacteria | Proteobacteria | Alphaproteobacteria | Rhodospirillales | uncultured |  |
| OTU199 | Bacteria | Proteobacteria | Deltaproteobacteria | Desulfovibrionales | Desulfovibrionaceae | Bilophila |
| OTU200 | Bacteria | Proteobacteria | Deltaproteobacteria | Desulfovibrionales | Desulfovibrionaceae | Desulfovibrio |
| OTU201 | Bacteria | Proteobacteria | Deltaproteobacteria | Desulfovibrionales | Desulfovibrionaceae | Mailhella |
| OTU202 | Bacteria | Proteobacteria | Deltaproteobacteria | Desulfovibrionales | Desulfovibrionaceae | uncultured |
| OTU203 | Bacteria | Proteobacteria | Gammaproteobacteria | Aeromonadales | Succinivibrionaceae | Succinivibrio |
| OTU204 | Bacteria | Proteobacteria | Gammaproteobacteria | Betaproteobacteriales | Burkholderiaceae | Parasutterella |
| OTU205 | Bacteria | Proteobacteria | Gammaproteobacteria | Betaproteobacteriales | Burkholderiaceae | Sutterella |
| OTU206 | Bacteria | Proteobacteria | Gammaproteobacteria | Betaproteobacteriales | Burkholderiaceae |  |
| OTU207 | Bacteria | Proteobacteria | Gammaproteobacteria | Enterobacteriales | Enterobacteriaceae | Escherichia-Shigella |
| OTU208 | Bacteria | Proteobacteria | Gammaproteobacteria | Enterobacteriales | Enterobacteriaceae | Hafnia-Obesumbacterium |
| OTU209 | Bacteria | Proteobacteria | Gammaproteobacteria | Enterobacteriales | Enterobacteriaceae | Proteus |
| OTU210 | Bacteria | Proteobacteria | Gammaproteobacteria | Enterobacteriales | Enterobacteriaceae |  |
| OTU211 | Bacteria | Proteobacteria | Gammaproteobacteria | Pasteurellales | Pasteurellaceae | Haemophilus |
| OTU212 | Bacteria | Proteobacteria | Gammaproteobacteria | Pseudomonadales | Pseudomonadaceae | Pseudomonas |
| OTU213 | Bacteria | Tenericutes | Mollicutes | Anaeroplasmatales | Anaeroplasmataceae | Anaeroplasma |
| OTU214 | Bacteria | Tenericutes | Mollicutes | Izimaplasmatales | gut metagenome | gut metagenome |
| OTU215 | Bacteria | Tenericutes | Mollicutes | Izimaplasmatales | uncultured bacterium | uncultured bacterium |
| OTU216 | Bacteria | Tenericutes | Mollicutes | Izimaplasmatales | uncultured organism | uncultured organism |
| OTU217 | Bacteria | Tenericutes | Mollicutes | Mollicutes RF39 | gut metagenome | gut metagenome |
| OTU218 | Bacteria | Tenericutes | Mollicutes | Mollicutes RF39 | metagenome | metagenome |
| OTU219 | Bacteria | Tenericutes | Mollicutes | Mollicutes RF39 | uncultured Mollicutes bacterium | uncultured Mollicutes bacterium |
| OTU220 | Bacteria | Tenericutes | Mollicutes | Mollicutes RF39 | uncultured bacterium | uncultured bacterium |
| OTU221 | Bacteria | Tenericutes | Mollicutes | Mollicutes RF39 | uncultured rumen bacterium | uncultured rumen bacterium |
| OTU222 | Bacteria | Tenericutes | Mollicutes | Mollicutes RF39 |  |  |
| OTU223 | Bacteria | Verrucomicrobia | Verrucomicrobiae | Opitutales | Puniceicoccaceae | uncultured |
| OTU224 | Bacteria | Verrucomicrobia | Verrucomicrobiae | Verrucomicrobiales | Akkermansiaceae | Akkermansia |
| OTU225 | Eukaryota | SAR | Stramenopiles | Incertae Sedis | Blastocystis | Blastocystis hominis |

Supplementary Table 4. Overview of Operational Taxonomic Units (OTU) obtained during 16S ribosomal RNA sequencing.

Supplementary Table 5A. Overview of selected proteins per comparison and corresponding metabolic pathways

| **Gene symbol** | **Uniprot accesion ID** | **HGNC Gene name** | **Protein interactions** | **Biological processes (6B)** |
| --- | --- | --- | --- | --- |
| ***Colorectal cancer versus controls*** | | | | |
| SIAE | Q9HAT2 | Sialate O-acetylesterase | Catalyses ester groups from sialic acid |  |
| HP | P00738 | Haptoglobin | Haemolysis, captures and combines with free plasma haemoglobin to allow hepatic recycling of heme iron and prevent kidney damage. Also acts as antimicrobial, antioxidant and antibacterial, modulator of acute phase response | 3,4,5,9,13 |
| CDHR5 | Q9HBB8-2 | Cadherin-related family member 5 | Intermicrovillar adhesion molecule, central role in differentiation of microvilli and epithelial brush border |  |
| HBB | P68871 | Haemoglobin subunit beta | Oxygen transport from lung to peripheral tissue | 2,3,4,5,9,10,14 |
| C3 | P01024 | Complement C3 | Activation of classical and alternative complement pathways | 1,6,7,10,11,14 |
| CP | P00450 | Ceruloplasmin | Copper-binding glycoprotein, iron transportation |  |
| SERPINA3 | P01011 | Alpha-1-antichymotrypsin;Alpha-1-antichymotrypsin His-Pro-less | Serpin peptidase inhibitor, unclear function, inhibits mast cell chymase | 1,6,12,14 |
| HBA1 | P69905 | Haemoglobin subunit alpha | Oxygen transport from lung to peripheral tissue | 2,3,4,5,8,9,13 |
| ***Adenomas versus controls*** | | | | |
| GUSB | P08236-3 | Beta-glucuronidase | Degradation of sulphates |  |
| HBB | P68871 | Haemoglobin subunit beta | Oxygen transport from lung to peripheral tissue | 2,3,4,5,9,10,14 |
| A2M | P01023 | Alpha-2-macroglobulin | Inhibition of proteinasen |  |
| HBA1 | P69905 | Haemoglobin subunit alpha | Oxygen transport from lung to peripheral tissue | 2,3,4,5,9,10,14 |
| ***Colorectal cancer versus adenomas*** | | | | |
| HP | P00738 | Haptoglobin | Haemolysis, captures and combines with free plasma haemoglobin to allow hepatic recycling of heme iron and prevent kidney damage. Also acts as antimicrobial, antioxidant and antibacterial, modulator of acute phase response | 3,4,5,9,13 |
| CEACAM8 | P31997 | Carcinoembryonic antigen-related cell adhesion molecule 8 | Glycoprotein member of CEA family |  |
| PRSS8 | Q16651 | Prostasin | Trypsin-like cleavage specificity with preference for poly-basic substrates. Serine protease. Stimulates epithelium sodium channel activity. |  |
| MUC2 | Q02817 | Mucin-2 | Coats epithelia of the intestines, airways and other mucus membrane-containing organs. Protective, lubricating barrier against particles and infectious agents at mucosal surface. | 7 |
| CP | P00450 | Ceruloplasmin | Copper-binding glycoprotein, iron transportation |  |
| SERPINA3 | P01011 | Alpha-1-antichymotrypsin;Alpha-1-antichymotrypsin His-Pro-less | Serpin peptidase inhibitor, unclear function, inhibits mast cell chymase | 1,7,13,15 |
| SGSH | P51688 | N-sulphoglucosamine sulphohydrolase | Catalyses a step in lysosomal heparan sulphate degradation |  |
| FCGBP | Q9Y6R7 | IgGFc-binding protein | Fc fragment of IgG binding protein |  |

Supplementary Table 5A. Selected proteins and their corresponding uniprot accession identification number, HUGO Gene Nomenclature Committee (HGNC) gene names, description of protein interaction and corresponding biological processes. Protein interaction and biological processes are based on the ©STRING consortium database and Database for Annotation, Visualization and Integrated Discovery (DAVID).

Supplementary Table 5B. List of biological processes corresponding with selected proteins

| **Number** | **Biological processes** |
| --- | --- |
| 1 | Negative regulation of endopeptidase activity |
| 2 | Oxygen transportation |
| 3 | Positive regulation of cell death |
| 4 | Response to hydrogen peroxide |
| 5 | Cellular oxidant detoxification |
| 6 | Positive regulation of angiogenesis |
| 7 | Maintenance of gastro-intestinal epithelium |
| 8 | Complement activation (alternative pathway) |
| 9 | Receptor mediated endocytosis |
| 10 | Hydrogen peroxide catabolic processes |
| 11 | Positive regulation of vascular endothelial growth factor production |
| 12 | Regulation of complement activation |
| 13 | Acute-phase response |
| 14 | Bicarbonate transport |
| 15 | Inflammatory response |

Supplementary Table 5B. List of biological processes corresponding with selected proteins in the current study.

Supplementary Table 6. Fold change of proteins for differentiation between colorectal cancer and controls

| **Proteins (Gene Symbol)** | **Fold Change** | **log2(FC)** |
| --- | --- | --- |
| HP | 44·292 | 5·469 |
| CP | 28·192 | 48·172 |
| HPX | 24·402 | 46·089 |
| HBDHBE1 | 22·509 | 44·924 |
| C3 | 19·079 | 42·539 |
| TF | 16·158 | 40·142 |
| C4A | 16·092 | 40·083 |
| A2M | 12·807 | 36·789 |
| FN1 | 12·094 | 35·962 |
| HBB | 10·925 | 34·495 |
| HBA1 | 93·806 | 32·297 |
| GC | 86·884 | 31·191 |
| FGG | 86·616 | 31·146 |
| FGB | 76·269 | 29·311 |
| CAT | 65·856 | 27·193 |
| PRDX2 | 55·684 | 24·773 |
| SERPINF2 | 53·878 | 24·297 |
| A1BG | 51·352 | 23·604 |
| SERPING1 | 50·946 | 2·349 |
| SOD1 | 0·20353 | -22·967 |
| SERPINB10 | 48·702 | 2·284 |
| MPO | 48·199 | 2·269 |
| MGAM2 | 0·21397 | -22·245 |
| FMN1 | 0·21468 | -22·197 |
| IGHV3-15 | 0·22272 | -21·667 |
| ELANE | 44·597 | 21·569 |
| TFF2 | 0·23063 | -21·163 |
| RBP4 | 41·721 | 20·608 |
| FLNB | 0·24695 | -20·177 |
| LGALS4 | 0·25407 | -19·767 |
| LGALS3 | 0·25839 | -19·524 |
| FOLH1FOLH1BNAALAD2 | 0·25843 | -19·522 |
| LTF | 3·731 | 18·996 |
| IGHV3-72 | 0·27465 | -18·643 |
| ACTN4ACTN2ACTN3 | 0·27787 | -18·475 |
| ALB | 35·455 | 1·826 |
| FBN1 | 0·30109 | -17·317 |
| CTSH | 0·30454 | -17·153 |
| CA2 | 32·832 | 17·151 |
| C5 | 32·678 | 17·083 |
| CDHR5 | 0·31148 | -16·828 |
| CELA2ACELA2B | 0·31586 | -16·627 |
| ADA | 0·31658 | -16·594 |
| CKB | 0·32045 | -16·418 |
| IGHV3-49 | 0·32258 | -16·323 |
| TFF3 | 0·32607 | -16·167 |
| LCT | 0·32847 | -16·062 |
| MDH2 | 0·34001 | -15·563 |
| NPC2 | 0·34087 | -15·527 |
| TPI1 | 0·35193 | -15·066 |
| SQOR | 0·356 | -14·901 |
| PRTN3 | 27·885 | 14·795 |
| BLVRB | 27·604 | 14·649 |
| IGHV3-33IGHV3-66IGHV3-53 | 0·36722 | -14·453 |
| LCP1 | 26·976 | 14·317 |
| COL4A1COL4A5 | 0·37485 | -14·156 |
| APOD | 26·615 | 14·123 |
| OLFM4 | 0·3768 | -14·081 |
| GPX3 | 26·442 | 14·028 |
| MCM3 | 0·37982 | -13·966 |
| QSOX1 | 0·38083 | -13·928 |
| PKMPKLR | 26·154 | 1·387 |
| IGHV4-28 | 0·38626 | -13·723 |
| HIST1H4A | 0·38938 | -13·608 |
| IDH1 | 0·39172 | -13·521 |
| AHCY | 25·113 | 13·285 |
| MYH3MYH2 | 0·39904 | -13·254 |
| SIAE | 0·40567 | -13·016 |
| MME | 0·40814 | -12·929 |
| IGKV3D-11IGKV3-11 | 0·40936 | -12·886 |
| MYH9MYH10MYH11 | 0·41234 | -12·781 |
| PIP | 24·091 | 12·685 |
| CDH17 | 0·41878 | -12·557 |
| EFCAB13 | 0·42113 | -12·476 |
| IGHV3-74IGHV7-4-1 | 0·43133 | -12·131 |
| HRG | 23·158 | 12·115 |
| LDHBLDHAL6ALDHC | 23·107 | 12·083 |
| CSTA | 0·43381 | -12·049 |
| CD177 | 22·978 | 12·002 |
| IGKV1-13IGKV1D-13 | 0·43654 | -11·958 |
| EPS8L3 | 0·43778 | -11·917 |
| REG1AREG1B | 0·44234 | -11·768 |
| ACE | 0·44334 | -11·735 |
| NPM1 | 0·44415 | -11·709 |
| ALDH2 | 0·44703 | -11·616 |
| GOT1 | 0·44829 | -11·575 |
| GALM | 0·45439 | -1·138 |
| SERPINA6 | 21·864 | 11·286 |
| PGLYRP1 | 21·724 | 11·193 |
| MUC12 | 0·46051 | -11·187 |
| SMPDL3A | 0·46057 | -11·185 |
| IGKV1-33IGKV1D-33 | 0·46784 | -10·959 |
| ARHGAP29 | 21·283 | 10·897 |
| LXN | 0·47298 | -10·802 |
| IGHV3-13 | 0·47324 | -10·794 |
| KRT19KRT15 | 0·47326 | -10·793 |
| BLMH | 21·099 | 10·772 |
| DNASE2 | 0·47755 | -10·663 |
| SLC4A1 | 20·802 | 10·568 |
| ALAD | 20·802 | 10·568 |
| PEPD | 0·48912 | -10·317 |
| ITIH2 | 20·387 | 10·276 |
| PEBP1 | 0·49111 | -10·259 |
| IGKV3-15IGKV3D-7 | 0·49234 | -10·223 |
| GAA | 0·49602 | -10·115 |
| GOT2 | 0·49719 | -10·081 |
| ATRN | 0·49864 | -10·039 |

Supplementary table 6. Fold change of proteins for the comparison between colorectal cancer and controls. A total of 7 proteins were differentially expressed. These were SIAE, HP, CDHR5, C3, SERPINF2 and HBA1.

Supplementary Table 7. Fold change proteins for differentiation between adenomas and controls

| **Proteins** | **Fold Change** | **log2(FC)** |
| --- | --- | --- |
| HP | 7·1525 | 2·8384 |
| TF | 6·2465 | 2·643 |
| LGALS3 | 0·18558 | -2·4299 |
| C3 | 4·761 | 2·2513 |
| CP | 4·3422 | 2·1184 |
| ACTN4ACTN2ACTN3 | 0·24249 | -2·044 |
| LGALS4 | 0·2447 | -2·0309 |
| HPX | 3·9439 | 1·9796 |
| HBB | 3·8549 | 1·9467 |
| MDH2 | 0·26412 | -1·9207 |
| A2M | 3·5508 | 1·8281 |
| GUSB | 3·3019 | 1·7233 |
| HBA1 | 2·9665 | 1·5688 |
| PEBP1 | 0·34029 | -1·5552 |
| KRT8 | 0·34466 | -1·5367 |
| CD9 | 0·37477 | -1·4159 |
| QSOX1 | 0·38115 | -1·3916 |
| SQOR | 0·38211 | -1·388 |
| ACADVL | 0·3838 | -1·3816 |
| TFF2 | 0·38482 | -1·3777 |
| HBDHBE1 | 2·5543 | 1·3529 |
| FLNB | 0·39419 | -1·343 |
| ALPL | 2·5269 | 1·3374 |
| MCM3 | 0·40751 | -1·2951 |
| REG1AREG1B | 0·43581 | -1·1982 |
| MYH9MYH10MYH11 | 0·44097 | -1·1812 |
| CPA2 | 2·2605 | 1·1766 |
| C4A | 2·2571 | 1·1745 |
| PGAM1PGAM2 | 2·244 | 1·1661 |
| IGHV3-15 | 0·45305 | -1·1422 |
| KRT13 | 0·45842 | -1·1253 |
| MUC13 | 0·45869 | -1·1244 |
| EPS8L3 | 0·46587 | -1·102 |
| FMN1 | 0·46819 | -1·0948 |
| PLS1 | 0·46958 | -1·0906 |
| SERPINF2 | 2·1254 | 1·0878 |
| CKMT1ACKMT2 | 2·1074 | 1·0755 |
| ALDH2 | 0·47526 | -1·0732 |
| TSPAN1 | 0·48175 | -1·0536 |
| GGH | 2·0532 | 1·0379 |
| SGSH | 2·0214 | 1·0153 |

Supplementary Table 7. Fold change of proteins for the comparison between adenomas and controls. No proteins were significantly differentially expressed between groups.

Supplementary Table 8. Fold change of proteins for comparison between colorectal cancer and adenomas

| **Proteins** | **Fold Change** | **log2(FC)** |
| --- | --- | --- |
| HBDHBE1 | 8·1662 | 3·0297 |
| FGB | 7·6269 | 2·9311 |
| FN1 | 7·4655 | 2·9002 |
| C4A | 6·668 | 2·7372 |
| CP | 6·3332 | 2·6629 |
| PRDX2 | 6·0862 | 2·6055 |
| HP | 5·9569 | 2·5746 |
| CAT | 5·7562 | 2·5251 |
| HPX | 5·6672 | 2·5026 |
| FGG | 5·1567 | 2·3664 |
| FOLH1FOLH1BNAALAD2 | 0·19442 | -2·3628 |
| A1BG | 5·1352 | 2·3604 |
| GC | 4·613 | 2·2057 |
| SERPING1 | 4·2564 | 2·0896 |
| C3 | 3·9893 | 1·9961 |
| SGSH | 0·25158 | -1·9909 |
| SERPINB10 | 3·9746 | 1·9908 |
| MGAM2 | 0·26385 | -1·9222 |
| A2M | 3·5841 | 1·8416 |
| MPO | 3·3854 | 1·7593 |
| CA2 | 3·2184 | 1·6863 |
| HBA1 | 3·1594 | 1·6596 |
| APOD | 3·1366 | 1·6492 |
| SOD1 | 0·35003 | -1·5144 |
| HBB | 2·8332 | 1·5024 |
| IGHV3-72 | 0·356 | -1·49 |
| CEACAM8 | 2·7912 | 1·4809 |
| C5 | 2·7677 | 1·4687 |
| BLVRB | 2·7604 | 1·4649 |
| GOT1 | 0·36568 | -1·4513 |
| CD9 | 2·6985 | 1·4321 |
| NPM1 | 0·3734 | -1·4212 |
| CST1CST4 | 0·37882 | -1·4004 |
| PKMPKLR | 2·6154 | 1·387 |
| IGHV3-33IGHV3-66IGHV3-53 | 0·38339 | -1·3831 |
| CTSH | 0·38631 | -1·3722 |
| LTF | 2·5511 | 1·3511 |
| TF | 2·5487 | 1·3498 |
| COL4A1COL4A5 | 0·40635 | -1·2992 |
| HIST1H4A | 0·40701 | -1·2968 |
| ADA | 0·40865 | -1·2911 |
| MYH3MYH2 | 0·41239 | -1·2779 |
| IGHV3-64D | 0·41334 | -1·2746 |
| ACE2 | 0·41593 | -1·2656 |
| SERPINF2 | 2·3894 | 1·2566 |
| MME | 0·42033 | -1·2504 |
| CSTB | 0·4208 | -1·2488 |
| LDHBLDHAL6ALDHC | 2·3648 | 1·2417 |
| TFF3 | 0·42484 | -1·235 |
| APCS | 2·3171 | 1·2123 |
| REG3A | 2·3162 | 1·2118 |
| HRG | 2·3158 | 1·2115 |
| ATP5F1B | 0·43276 | -1·2084 |
| CA1 | 2·2634 | 1·1785 |
| LCP1 | 2·2514 | 1·1708 |
| CSTA | 0·44454 | -1·1696 |
| PRTN3 | 2·2319 | 1·1582 |
| FBN1 | 0·44837 | -1·1572 |
| HLA-DRB1 | 0·45289 | -1·1428 |
| RBP4 | 2·2062 | 1·1416 |
| DNPEP | 0·45667 | -1·1308 |
| SERPINA6 | 2·1864 | 1·1286 |
| PGLYRP1 | 2·1571 | 1·1091 |
| RNASE3 | 0·4641 | -1·1075 |
| TKT | 2·1415 | 1·0986 |
| ELANE | 2·1372 | 1·0957 |
| AKAP9 | 0·47175 | -1·0839 |
| FMN1 | 0·47395 | -1·0772 |
| SLC4A1 | 2·0802 | 1·0568 |
| ALAD | 2·0802 | 1·0568 |
| CDHR5 | 0·48456 | -1·0453 |
| AMY1A | 2·0581 | 1·0413 |
| GAA | 0·48782 | -1·0356 |
| FTH1 | 0·49194 | -1·0235 |
| GPX3 | 2·0327 | 1·0234 |
| MUC1 | 2·0315 | 1·0225 |
| AHCY | 2·012 | 1·0086 |
| PRSS8 | 0·4993 | -1·002 |

Supplementary Table 8. Fold change of proteins for the comparison between colorectal cancer and adenomas. No proteins were significantly differentially expressed between groups.
